# Sterol-regulated transmembrane protein TMEM86a couples LXR signaling to regulation of lysoplasmalogens in macrophages

**DOI:** 10.1016/j.jlr.2022.100325

**Published:** 2022-12-31

**Authors:** Suzanne A.E. van Wouw, Marlene van den Berg, Maroua El Ouraoui, Amber Meurs, Jenina Kingma, Roelof Ottenhoff, Melanie Loix, Marten A. Hoeksema, Koen Prange, Gerard Pasterkamp, Jerome J.A. Hendriks, Jeroen F.J. Bogie, Jan B. van Klinken, Frederic M. Vaz, Aldo Jongejan, Menno P.J. de Winther, Noam Zelcer

**Affiliations:** 1 Department of Medical Biochemistry, Amsterdam UMC, Amsterdam Institutes of Cardiovascular Sciences, Infection and Immunity, and Gastroenterology Endocrinology and Metabolism, University of Amsterdam, Amsterdam, the Netherlands; 2 Department of Immunology and Infection, Biomedical Research Institute, Hasselt University, Diepenbeek, Belgium; 3 Department of Experimental Cardiology, Utrecht UMC, Utrecht, the Netherlands; 4 Amsterdam UMC location University of Amsterdam, Department of Clinical Chemistry and Pediatrics, Laboratory Genetic Metabolic Diseases, Emma Children's Hospital, Amsterdam, the Netherlands; 5 Core Facility Metabolomics, Amsterdam UMC location University of Amsterdam, Amsterdam, the Netherlands; 6 Department of Human Genetics, Leiden University Medical Center, Leiden, the Netherlands; 7 Department of Epidemiology and Data Science, Bioinformatics Laboratory, of Academic Medical Center, University of Amsterdam, Amsterdam, the Netherlands

**Keywords:** bone marrow–derived macrophages, plasmalogens, lysoplasmalogens, LXR, lipid metabolism, lipidomics, sterol, atherosclerotic plaques, transcriptional regulation, membrane fluidity, ABCA1, ATP Binding Cassette Subfamily A Member 1, AcLDL, Acetylated LDL, ADH, Alcohol dehydrogenase, BMDM, Bone marrow–derived macrophages, GW, GW3965, LCM, L929-conditioned medium, LPC, 1-acyl lysophosphatidylcholine, LPC [O], 1-O-alkyl lysophosphatidylcholine, LPC', 1-O-alkenyl lysophosphatidylcholine, LPCAT3, lysophosphatidylcholine acyltransferase 3, LPE, 1-acyl lysophosphatidylethanolamine, LPE [O], 1-O-alkyl lysophosphatidylethanolamine, LPE', 1-O-alkenyl lysophosphatidylethanolamine, LXR, Liver X Receptor, PBMC, Human peripheral mononuclear blood cells, PC, 1-acyl phosphatidylethanolamine, PC', 1-O-alkenyl phosphatidylcholine, PE, 1-acyl phosphatidylcholine, PE', 1-O-alkenyl phosphatidylethanolamine, PM, Peritoneal Macrophage, PUFA, polyunsaturated fatty acids, T09, T0901317, TMEM86a, Transmembrane protein 86a

## Abstract

Lysoplasmalogens are a class of vinyl ether bioactive lipids that have a central role in plasmalogen metabolism and membrane fluidity. The liver X receptor (LXR) transcription factors are important determinants of cellular lipid homeostasis owing to their ability to regulate cholesterol and fatty acid metabolism. However, their role in governing the composition of lipid species such as lysoplasmalogens in cellular membranes is less well studied. Here, we mapped the lipidome of bone marrow–derived macrophages (BMDMs) following LXR activation. We found a marked reduction in the levels of lysoplasmalogen species in the absence of changes in the levels of plasmalogens themselves. Transcriptional profiling of LXR-activated macrophages identified the gene encoding transmembrane protein 86a (TMEM86a), an integral endoplasmic reticulum protein, as a previously uncharacterized sterol-regulated gene. We demonstrate that *TMEM86a* is a direct transcriptional target of LXR in macrophages and microglia and that it is highly expressed in TREM2^+^/lipid-associated macrophages in human atherosclerotic plaques, where its expression positively correlates with other LXR-regulated genes. We further show that both murine and human TMEM86a display active lysoplasmalogenase activity that can be abrogated by inactivating mutations in the predicted catalytic site. Consequently, we demonstrate that overexpression of *Tmem86a* in BMDM markedly reduces lysoplasmalogen abundance and membrane fluidity, while reciprocally, silencing of *Tmem86a* increases basal lysoplasmalogen levels and abrogates the LXR-dependent reduction of this lipid species. Collectively, our findings implicate TMEM86a as a sterol-regulated lysoplasmalogenase in macrophages that contributes to sterol-dependent membrane remodeling.

## Graphical abstract

**Figure:**
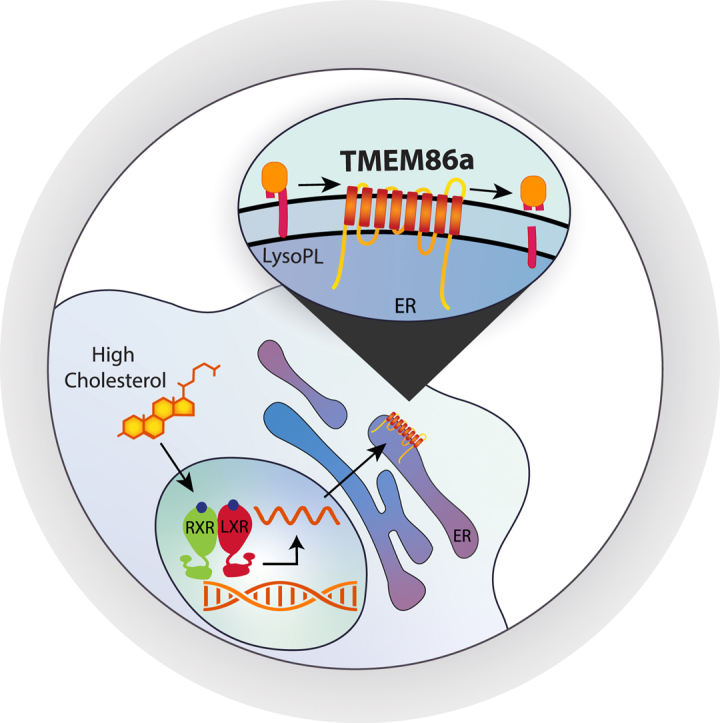

The sterol-responsive liver X receptors (LXRs) α and β transcription factors (*NR1H3* and *NR1H2*, respectively) are members of the large family of ligand-activated nuclear receptors (1). Their endogenous ligands are oxysterols and intermediates of the cholesterol biosynthetic pathway (1, 2). Upon ligand engagement, LXRs induce the expression of a wide set of transcriptionally responsive genes that act primarily to promote cholesterol efflux (3, 4), limit cholesterol uptake (5, 6), and enhance fatty acid synthesis (7, 8). As such, LXR signaling is an important determinant of cellular and whole-body lipid homeostasis, and consequently, their activity has been implicated in a wide range of human conditions, including cardiovascular, inflammatory, and neurodegenerative diseases (9, 10). In macrophages, LXR signaling is critical owing to the need of these cells to cope and adapt to a high and dynamic flux of lipids from (modified) lipoproteins and efferocytosis of apoptotic cells.

In recent years, it has become apparent that next to their direct role in cholesterol metabolism, LXRs also govern remodeling of membrane composition and that this in turn has profound consequences on, e.g., cholesterol synthesis and transport (11). A prime example of this is the transcriptional regulation of lysophosphatidylcholine acyltransferase 3 (*MBOAT5/LPCAT3*) by LXRs in a variety of cell types (12, 13, 14, 15). LPCAT3 is an ER-resident acyltransferase that catalyzes the incorporation of a preferably polyunsaturated fatty acid (PUFA), at the *sn-2* position of various classes of lysophospholipids, the preferred substrates being 1-acyl lysophosphatidylcholine (LPC) and to a lesser extent 1-acyl lysophosphatidylethanolamine (LPE) (13, 16, 17, 18). The effect of LXR on other lipid species is less well understood.

In addition to nonether phospholipids, plasmalogens are an abundant class of ethanolamine and choline ether glycerophospholipids, which uniquely carry an *sn-1* vinyl ether-linked fatty alcohol (19, 20). The backbone of plasmalogens is synthesized in peroxisomes and subsequently remodeled in the endoplasmic reticulum of all cells. Their abundance varies among cell types and in most cells typically constitute ∼20% of cell membrane phospholipids. Yet in some organ systems, such as the heart, brain, and immune system, plasmalogen levels are higher and represent a higher fraction of total phospholipids (reviewed in (20)). Plasmalogens are important for maintaining membrane rigidity (19, 21), and have been implicated in distinct signaling pathways (22) and as antioxidants (23, 24). They have also been linked to inflammatory responses, as phospholipase A2-mediated hydrolysis to lysoplasmalogen (1-O-(1Z-alkenyl)-2-lyso-sn-GPEth(Cho)) releases the *sn-2*-associated PUFA, which is a rich source for bioactive lipid mediators (25, 26, 27). Given their broad distribution, disturbed plasmalogen metabolism has been linked to the development of, amongst others, cancer and cardiovascular and neurodegenerative disease in humans (20, 28). Remarkably, in contrast to the biosynthesis of plasmalogens, the regulation of their catabolism and the fate of lysoplasmalogens (1-O-alkenyl lysophosphatidylethanolamine [LPE’] and LPC’) is less well understood. Recent advances in lipidomic and transcriptomic analysis empower unbiased interrogation of lipid metabolism in cells. Using a multiomic approach, we mapped the lipidomic landscape of LXR-activated macrophages. We report here that LXR activation is an important determinant of lysoplasmalogen catabolism in macrophages and microglia and identify transmembrane protein 86a (TMEM86a) as a novel sterol-inducible lysoplasmalogenase in these cells.

## MATERIALS AND METHODS

### Cell culture

#### Isolation of bone marrow–derived macrophages and thioglycolate-induced peritoneal macrophages

Murine bone marrow–derived macrophages (BMDMs) were isolated from femurs and tibias of wild-type C57BL/6J mice, as previously reported (29). Isolated cells were cultured in RPMI-1640 (Gibco), 10% fetal calf serum (FCS), penicillin (100U/ml), streptomycin (100 μg/ml) (Gibco) supplemented with 15% L929-conditioned medium (LCM) for 7 days. Fresh medium was added on day 4, and the medium was replaced on day 6. Experiments were conducted on day 8 postisolation. Peritoneal macrophages (PMs) were isolated as described previously (5). Adherent cells were cultured in RPMI-1640 containing (Gibco), 10% FCS, penicillin (100U/ml), and streptomycin (100 μg/ml) (Gibco) and used for experiments within 24 h. All mouse experiments were approved by the Committee for Animal Welfare (University of Amsterdam).

#### Isolation of murine microglia

Microglia were isolated as described previously (30). Briefly, forebrains from postnatal P1-P3 C57BL/6J pups were isolated and incubated in L15 Leibovitz medium (Gibco) containing 1:10 Trypsin (Sigma-Aldrich) for 15 min at 37°C, after which DMEM high glucose (Invitrogen) supplemented with 10% FCS, 50U/ml penicillin, and 50U/ml streptomycin, (DMEM 10:1 medium) was added. Cells were separated using trituration with serum-coated Pasteur pipettes (Sigma-Aldrich), passed through a 70 μm cell strainer, flushed, and spun down at 170 g for 10 min. Subsequently, isolated cells were plated at a density of 2 forebrains/75cm2 flask and grown to 80% confluence (∼6 days) when complete DMEM supplemented with 30% LCM was added. Following 6 additional days of differentiation, microglia were isolated using the shake-off procedure and plated at a density of 250,000 cells/well (24 well plate) in complete DMEM supplemented with 15% LCM.

#### Isolation of human peripheral mononuclear blood cells

Peripheral mononuclear blood cells (PBMCs) were isolated as described previously (31)*.* Briefly, blood was collected from healthy human donors, and EDTA-plasma was separated using centrifugation at 400 *g* for 10 min. The remaining blood fraction was diluted with 1x PBS containing 2 mM EDTA without Ca^2+^ and Mg^2+^ (Lonza). A Ficoll-paque (GE Healthcare Life Sciences) density gradient separation and centrifugation at 400 *g* for 20 min was used to isolate the layer of PBMCs. Collected cells were washed twice in PBS with 2 mM EDTA, and CD14^+^ monocytes were separated using EasySep™ (Stemcell Technologies). Cells were plated in 24-well plates at a density of 0.5 ×10^6^ cells per well in RPMI 1640 containing penicillin (100U/ml), streptomycin (100 μg/ml), 1% nonessential amino acids, 2.5% FCS, and 1% sodium pyruvate.

#### LXR activation in cells

Isolated cells were treated with agonists to stimulate LXR-dependent signaling. GW3965 (GW) (Sigma) was used at a concentration of 1 or 2 μM for 6 h for RNA isolation and 16 h for protein collection and lipidomic profiling. 22R-hydroxycholesterol was dissolved in ethanol and used at a concentration of 5 μM for 16 h in combination with sterol-depleted medium [RPMI with 10% lipoprotein-deficient serum, penicillin (100U/ml), streptomycin (100ug/ml) (Gibco), 2.5 μg/ml simvastatin (Calbiochem), and 100 μM mevalonic acid (Sigma)]. T0901317 (T09) (Cayman Chemical) was used at a concentration of 10 μM for 24 h. Acetylated LDL (AcLDL) (Kalen Biochemicals) was added to the culture medium at a final concentration of 50 μg/ml for 24 h.

#### Generation of lentiviral Tmem86a constructs and viral transduction of BMDMs

*Tmem86a* expression was either silenced or increased using lentiviral transduction. Briefly, to silence *Tmem86a,* four independent shRNA sequences were designed using DSIR (32), cloned into pENTR/pTER+ (430-1) (Addgene #17453), and recombined into pLentiX1-PGK-GFP-DEST (694-6, modified from Addgene #17297). All plasmids used in this study are listed in supplemental Table S1. The two shRNAs showing the most effective *Tmem86a* silencing in pilot experiments were selected for further use. A scrambled shRNA was used as control in these experiments. The sequence of shRNAs used is shown in supplemental Table S2. For overexpression, m*Tmem86a* cDNA was amplified from BMDMs, cloned into pDONR221 with the indicated tags, and recombined into pLenti6.3/T0/V5-DEST. The correctness of all generated constructs was verified by sequencing. Lentiviral particles were produced in HEK293T cells as previously described (33). The culture medium containing lentiviral particles was concentrated 10-fold by ultracentrifugation at 20.000 *g* for 2 h at 4°C. BMDMs were transduced with concentrated lentiviral particles on day 2 of differentiation (1:7.5 v/v), a procedure that was experimentally tested to give optimal transduction efficiency. The membrane topology of Tmem86a was predicted using TOPCONS (34), which incorporates five distinct algorithms in its output.

#### Pharmacological dosing of mice with GW

Male wild-type C57BL6/N mice (n = 5/group) were administered 40 mg/kg/day GW (Axon MedChem, Axon 1266) or vehicle (0.5% methylcellulose) twice daily by oral gavage for 4.5 days. Following the last dose, mice were fasted for 4 h prior to sacrifice. Tissues were collected for RNA isolation.

#### Quantitative PCR

RNA was isolated from tissues by manual Tri reagent (Sigma) extraction following the manufacturer's guidelines. RNA from cells was extracted and isolated using the Direct-zol RNA miniprep kit (Zymo Research). iScript reverse transcription reagent (Bio-Rad) was used to generate cDNA, and SensiFAST SYBR (Bioline) was used for real-time quantitative PCR (RT-qPCR). A LightCycler 480 II system (Roche) was used to perform the measurements, and gene expression was normalized to the expression levels of Rplp0/Rpl13. Oligonucleotide sequences used for qPCR are provided in supplemental Table S3.

#### Immunoblot analysis

Total cell lysates were prepared in radioimmunoprecipitation buffer (150 mM NaCl, 1% Nonidet P-40, 0.1% sodium deoxycholate, 0.1% SDS, 100 mM Tris-HCl, pH 7.4) supplemented with a cocktail of protease inhibitors (Roche) and 10 mM phenylmethylsulfonyl fluoride (Sigma). The lysates were cleared by centrifugation at 4 °C for 10 min at 10,000xg. Initial experiments showed aggregation of the TMEM86a protein and therefore 5% SDS was added to samples to counteract this unless otherwise indicated. Proteins were separated on NuPAGE Novex 4%–12% Bis-Tris gels and transferred to nitrocellulose membranes (ThermoFisher). All antibodies used are listed in supplemental Table S4. Secondary HRP-conjugated antibodies (Zymed) were used and visualized with chemiluminescence on a GE IQ800 (GE Healthcare). Unless indicated, blots shown are representative of at least three independent experiments with similar results. Signals were quantified using ImageJ software.

#### Immunofluorescence staining of BMDM

For immunofluorescence staining, BMDM cells were cultured on coverslips and differentiated as described above. After differentiation, cells were fixed for 10 min with 4% paraformaldehyde in PBS and subsequently permeabilized and blocked by incubation with 0.5% TritonX-100% and 0.25% BSA in PBS for 15 min. Antibodies for immunostainings were diluted in 0.5% Triton X-100% and 0.25% BSA in PBS, and the coverslips were incubated with primary antibodies for 1.5 h, followed by the secondary antibodies for 1 h, and then DAPI for 10 min. Between steps, coverslips were washed three times with 0.5% Triton in PBS. Slides were mounted using Mowiol4-88/DABCO solution (Calbiochem). All antibodies used are described in supplemental Table S4. Fluorescent imaging was done on a Confocal SP8-X DLS Lightsheet.

#### Lipidomic analysis

Lipidomic analysis of samples was performed at the Core Facility Metabolomics of the Amsterdam UMC, location AMC, Amsterdam, the Netherlands (www.cfmetabolomics.nl), and performed essentially as previously described (35). In a 2 ml tube, the following amounts of internal standards dissolved in 1:1 (v/v) methanol:chloroform were added to each sample which contained a protein equivalent of 1 mg of the cellular homogenate: Bis(monoacylglycero)phosphate BMP(14:0)_2_ (0.2 nmol), ceramide-1-phosphate C1P (d18:1/12:0) (0.125 nmol), D7-cholesteryl ester CE(16:0) (2.5 nmol), ceramide Cer(d18:1/12:0) (0.125 nmol), ceramide Cer(d18:1/25:0) (0.125 nmol), cardiolipin CL(14:0)_4_ (0.1 nmol), diacylglycerol DG(14:0)_2_ (0.5 nmol), glucosylceramide GlcCer(d18:1/12:0) (0.125 nmol), lactosylceramide LacCer(d18:1/12:0) (0.125 nmol), lysophosphatidic acid LPA(14:0) (0.1 nmol), lysophosphatidylcholine LPC(14:0) (0.5 nmol), lysophosphatidylethanolamine LPE(14:0) (0.1 nmol), lysophosphatidylglycerol LPG(14:0) (0.02 nmol), phosphatidic acid PA(14:0)_2_ (0.5 nmol), phosphatidylcholine PC(14:0)_2_ (2 nmol), phosphatidylethanolamine PE(14:0)_2_ (0.5 nmol), phosphatidylglycerol PG(14:0)_2_ (0.1 nmol), phosphatidylinositol PI(8:0)_2_ (0.5 nmol), phosphatidylserine PS(14:0)_2_ (5 nmol), sphinganine 1-phosphate S1P(d17:0) (0.125 nmol), sphinganine-1-phosphate S1P(d17:1) (0.125 nmol), ceramide phosphocholines SM(d18:1/12:0) (2.125 nmol), sphingosine SPH(d17:0) (0.125 nmol), sphingosine SPH(d17:1) (0.125 nmol), and triacylglycerol TAG(14:0)_2_ (0.5 nmol). All internal standards were purchased from Avanti Polar Lipids. After the addition of the internal standards, 1.5 ml 1:1 (v/v) methanol:chloroform was added before thorough mixing. The samples were then centrifuged for 10 min at 14.000 rpm, supernatant transferred to a glass vial and evaporated under a stream of nitrogen at 60°C. The residue was dissolved in 150 μl of 1:1 (v/v) methanol:chloroform. Lipids were analyzed using a Thermo Scientific Ultimate 3000 binary HPLC coupled to a Q Exactive Plus Orbitrap mass spectrometer. For normal phase separation, 5 μl of each sample was injected onto a Phenomenex® LUNA silica, 250 ∗ 2 mm, 5 μm 100 Å. Column temperature was held at 25°C. Mobile phase consisted of (A) 85:15 (v/v) methanol:water containing 0.0125% formic acid and 3.35 mmol/l ammonia and (B) 97:3 (v/v) chloroform:methanol containing 0.0125% formic acid. Using a flow rate of 0.3 ml/min, the LC gradient consisted of: isocratic at 10% A 0–1 min, ramp to 20% A at 4 min, ramp to 85% A at 12 min, ramp to 100% A at 12.1 min, isocratic at 100% A 12.1–14 min, ramp to 10% A at 14.1 min, isocratic at 10% A for 14.1–15 min. For reversed phase separation, 5 μl of each sample was injected onto a Waters HSS T3 column (150 − 2.1 mm, 1.8 μm particle size). Column temperature was held at 60°C. Mobile phase consisted of A 4:6 (v/v) methanol:water and B 1:9 (v/v) methanol:isopropanol, both containing 0.1% formic acid and 10 mmol/L ammonia. Using a flow rate of 0.4 ml/min, the LC gradient consisted of: isocratic at 100% A at 0 min, ramp to 80% A at 1 min, ramp to 0% A at 16 min, isocratic at 0% A for 16–20 min, ramp to 100% A at 20.1 min, isocratic at 100% A for 20.1–21 min. MS data were acquired using negative and positive ionization by continuous scanning over the range of m/z 150 to m/z 2000 at a resolution of 280,000 full width at half maximum. Data were analyzed using an in-house developed lipidomics pipeline written in the R programming language (http://www.r-project.org) and MATLAB. Lipid identification was based on a combination of accurate mass, (relative) retention times, fragmentation spectra (when required), analysis of samples with known metabolic defects, and the injection of relevant standards. Lipid classes are defined in our lipidomics pipeline in terms of their generic chemical formula, where R represents the radyl group. Upon import of the lipid database in the annotation pipeline, the generic chemical formula of each lipid class is expanded by replacing the R-element with a range of possible radyl group lengths and double bond numbers. The resulting expanded list of chemical formulas is then used to calculate the neutral monoisotopic mass of each species. The reported lipid abundances are semiquantitative and calculated by dividing the response of the analyte (area of the peak) by that of the corresponding internal standard multiplied by the concentration of that internal standard (arbitrary unit, A.U). The suffix [O] indicates lipids containing an alkyl-ether group, whereas the addition of a quote ‘ indicates an alkenyl-ether group. As no dedicated internal standard for ether lipids are available, we used the (L)PC/PE internal standards to normalize the corresponding ether lipid species as described above. Annotation of ether lipid species, more specifically the differentiation of alkyl-(L)PE/PC from alkenyl-PE/PC species, was performed based on stable (relative) retention time differences between corresponding alkyl- and alkenyl-species. The PC[O] and PE[O] species elute at approximately the same retention time on the reversed phase column as the 1-O-alkenyl phosphatidylcholine (PC’) and 1-O-alkenyl phosphatidylethanolamine (PE’) (alkenyl) species with the same fatty acyl composition, and significantly earlier than the PC’ and PE’ species with the same mass (data not shown). This reproducible difference was corroborated using a PEDS1 mouse model in which the enzyme that converts alkyl-species into alkenyl-species is deficient. In PEDS1 alkenyl-species are not detectable whereas alkyl-species accumulate (data not shown) confirming the correct annotation of alkyl- and alkenyl-species. The reported lipid abundances are semiquantitative and calculated by dividing the response of analyte by that of the corresponding internal standard multiplied by the concentration of that internal standard (arbitrary unit, A.U). Although this can be regarded as a concentration, we use arbitrary response as unit because the response of lipid species in the mass spectrometer varies considerably depending on their fatty acid composition, and hence their concentration can only be estimated by using a single internal standard per class.

#### RNAseq

RNA from BMDM was isolated with the Direct-zol RNA miniprep kit (Zymo Research) and quantified fluorometrically with a Qubit 3 Fluormeter (Invitrogen). The quality of the RNA was evaluated with a 2100 Bioanalyzer (Agilent Technologies). A KAPA mRNA hyperPrep kit (KAPA Biosystems) was used for mRNA library construction, and sequencing was performed on an Illumina HiSeq4000 at the Core Facility genomics of the Amsterdam UMC, location AMC, Amsterdam, the Netherlands. Bioinformatic analysis was performed in collaboration with the Bioinformatics Core Unit of the Amsterdam UMC, location AMC, Amsterdam, the Netherlands as previously described (36). Briefly, quality control (fastQC, dupRadar, Picard Tools) (37) was performed on the reads. Subsequently, they were trimmed using Trimmomatic v0.32 (38) and aligned to the mouse genome using HISAT2 (v2.1.0) (39). Counts were obtained using corresponding GTF and HTSeq (v0.11.0) (40). TMM (EdgeR) (41) and limma/voomR (42) packages were used to perform statistical analysis. Count data were transformed to log2-counts per million and normalized using the trimmed mean of M-values method (41). Voom was used to precision weigh the data. Bayes moderated *t* test was used to assess differential expression within limma’s linear model framework and the precision weights estimated by voom (42). The mouse ID was included as a random effect in the linear model to estimate the consensus correlation, using duplicateCorrelation from the limma package. The *P* values were corrected for multiple testing using the Benjamini-Hochberg false discovery rate. BiomaRt (release 94) was used to reannotate the genes. Results are shown in an in-house made Shiny-app. Analysis was performed using R v3.5.0 and Bioconductor v3.7.

#### ATACseq and ChiP-seq visualization

To visualize LXR binding to the *Tmem86a* enhancer publicly available ATAC-seq, H3K27Ac and LXR ChIP-seq datasets in mouse BMDM were used (43, 44). Data were downloaded from the GEO database (GSE15963and GSE79423) mapped to the mm10 genome using bowtie2 v2.2.9 (45) and tag directories and track hubs for the UCSC genome browser were made using HOMER v4.11 (46). To evaluate the LXR isoform-specific contribution to regulation of *Tmem86a,* we retrieved previously reported LXR ChIP-seq datasets (GSE104027) (47). This experiment compared immortalized BMDMs from LXRαβ^(-/-)^ mice that ectopically express 3xFLAG-tagged LXRα, 3xFLAG-LXRβ, or empty vector control (LXR double KO control). Cells were treated with 1 μM GW for 24 h, and LXRs were immunoprecipitated with anti-Flag M2 antibody (Sigma, F3165). Additionally, we also incorporated ChIP-seq data from Daniel *et al.* (48)*.* describing the genome-wide localization analyses of RXR in BMDM treated with GW (sequence read archive accession SRR1514119). The aforementioned ChIP-seq public data were mapped with Bowtie2 software to the mm10 assembly of the mouse genome. The resulting SAM output files were analyzed with HOMER software using recommended default settings. The result from each sequencing experiment was normalized to 1x10^7^ uniquely mapped tags. Sequencing experiments were visualized with the UCSC genome browser.

#### Visualization of human PBMC and mouse macrophage transcriptional datasets

Publicly available datasets were downloaded from the GEO database (GSE118656, BMDM from LXR wild-type and knockout mice (49); GSE90815 mouse thioglycolate-elicited PMs treated with LXR ligands (50); and GSE35967, human PBMC treated with LXR ligand (51)). Data were visualized using R2: Genomics Analysis and Visualization Platform (www.r2.amc.nl).

#### Single cell RNA-seq analysis of human carotid plaques

Single cell RNA-seq libraries were created from human carotid plaques as previously described (52). Count tables were loaded into an R 4.1.3 environment and analyzed with Seurat 4.1.0 (53) following the ‘SCTransform’ workflow (54). Plots were created using ggplot2 3.3.5 (55). Macrophage clusters were identified based on marker gene expression. Macrophages were binned according to *TMEM86a* expression as follows: Zero: no *TMEM86a* expression; low: bottom 25th percentile; medium: between 25th and 75th percentile; high: over 75th percentile of *TMEM86a* expression.

#### Lysoplasmalogenase assay

Crude membrane fractions were isolated from control and m/hTMEM86a-overproducing HEK293T cells in three independent experiments as previously reported (56), with minor modifications. Briefly, cells were washed, scraped in cold PBS, and centrifuged at 1000 *g* for 5 min at 4°C. The cell pellet was resuspended in membrane-isolation buffer (250 mM sucrose and 10 mM Tris/HCL pH 7.4 supplemented with protease inhibitors) and disrupted with 20 strokes in a Dounce homogenizer. Subsequently, the suspension was centrifuged at 1000 *g* for 5 min at 4°C, and the supernatant collected and centrifuged at 20000 *g* for 15 min at 4°C. Pelleted membranes were resuspended in membrane-isolation buffer, and the protein concentrations were measured using the BCA assay (Fisher). Equal amounts of protein were used for a lysoplasmalogenase assay following the procedure described by Jurkowitz *et al.* (57)*.* The standard reaction mixture contained 70 mM glycylglycine-NaOH (pH 7.1) (Sigma), 1 mM DTT (Sigma), 0.2 mM NADH (Roche), 250 IU/ml alcohol dehydrogenase (ADH) (Sigma), and 423 nmol/ml LPE’ (Brain lyso PE plasmalogens, Avanti) or LPC’ (1-O-1'-(Z)-octadecenyl-2-hydroxy-sn-glycero-3-phosphocholine, C18-(Plasm)-LPC,Avanti). Reactions were conducted in a total volume of 110 μl at 37°C, and NADH absorbance at 340 nm was continuously monitored on a synergy HT Plate Reader (Biotek). The decrease in absorbance during the first 15 min linear phase was calculated and is shown.

#### Membrane fluidity assay

Membrane fluidity was measured by determining the ratio of the monomer to excimer of pyrenedecanoic acid (PDA) following the manufacturers’ instructions (Abcam, ab189819). Briefly, BMDMs were cultured in wells of a 96 well-plate and treated as indicated. Cell were subsequently labeled in staining solution containing 5 μM PDA supplemented with 0.8% Pluronic F127 at 25°C for 1 h in the dark. Unincorporated PDA was removed by two consecutive washes with PBS, after which 50 μl medium was added. Fluorescence was measured with a CLARIOstar Plus (BMG Labtech) with excitation set to 350 nm and emission set to 400 nm (monomer) and 470 nm (excimer). Background fluorescence was measured in nonlabeled cells and subtracted from all samples. The normalized ratio between fluorescence of the monomer to excimer (470 nm/400 nm) was calculated and is plotted.

#### Statistical analysis

Data are presented as the mean ± standard error of the mean. Statistical analysis was performed using Graphpad Prism, version 9. Normality of data was tested using Shapiro-Wilk tests. If data were normally distributed, *t* test was used for single comparisons or ANOVA for multiple testing. When data were not normally distributed, the analysis was corrected by using Mann-Whitney as an alternative for the *t* test, and Kruskal-Wallis with Dunn’s multiple comparison test as an alternative to ANOVA. If assumptions for homogeneity of variance are not met, *t* test with Welch correction and ANOVA with Holm-Sidak post hoc analysis was used. A *P* < 0.05 was considered significant. ∗ *P*<0.05, ∗∗ *P*<0.005, ∗∗∗ *P*<0.0005.

## RESULTS

**Fig. 1:**
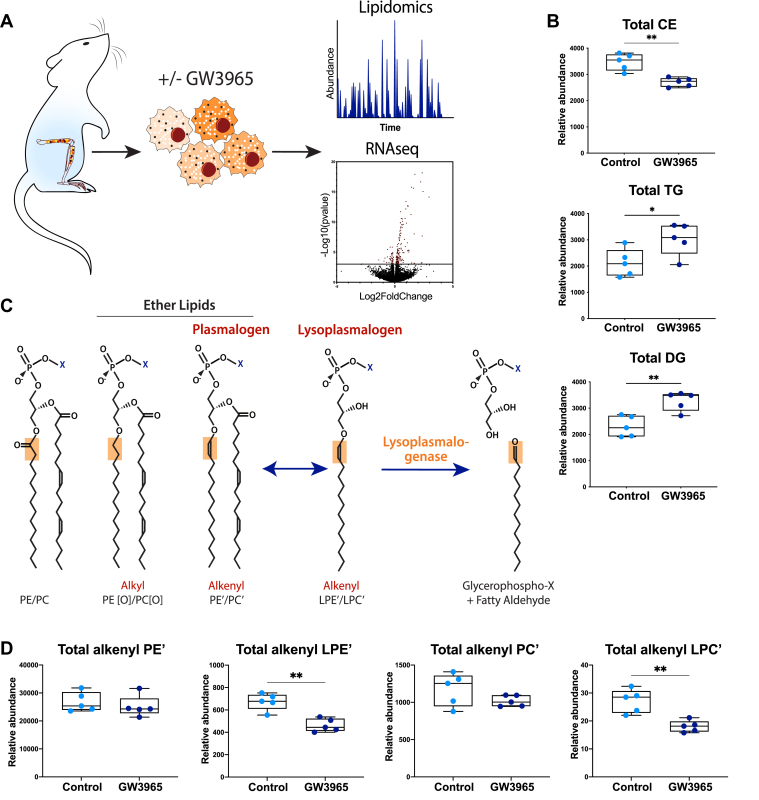
Decreased lysoplasmalogens in LXR-stimulated macrophages. A: A schematic overview of the experiment. BMDMs were isolated from wild-type mice (N = 5/group) and stimulated with 1 μM GW3965 (LXR agonist) or vehicle control for 6 h or 16 h for transcriptomic and lipidomics analysis, respectively. Lipid composition was measured using lipidomic profiling and transcriptional profiling was obtained by RNAseq. The total abundance of (B) cholesterol esters (CEs), triglycerides (TGs), and diglycerides (DGs) is shown. C: Lysoplasmalogens are a class of ether lipids uniquely containing an alkenyl bond at the *sn-1* position. Lysoplasmalogens are derived from plasmalogens after enzymatic release of the *sn-2* conjugated fatty acid. Release of the *sn-1* linked fatty aldehyde requires the specific activity of a lysoplasmalogenase. D: Alkenyl PE or PC (PE’ and PC’, respectively) and alkenyl lyso PE or PC (LPE’ and LPC’, respectively) were quantified and total amounts shown. Box plots show the mean ± SEM. ∗ *P*<0.05, ∗∗ *P*<0.01.

LXR signaling in macrophages is extensively studied due to the potential of harnessing this pathway in therapeutic strategies. To expand our understanding of LXR signaling in macrophages, we evaluated the lipidomic landscape of murine BMDM following pharmacological LXR activation with the synthetic ligand GW (Fig. 1A). Unbiased lipidomic analysis of LXR activation reported the levels of 1544 lipid species and derivatives and indicated that LXR activation had a profound effect on the lipidome landscape of these cells (supplemental Fig. S1A and supplemental Table S5). As anticipated, LXRs increased total cellular triacylglycerol and diacylglycerol levels, concordant with increased fatty acid synthesis (Fig. 1B). Additionally, this treatment decreased cellular cholesterol-ester content, which is consistent with mobilization of cholesterol towards cholesterol efflux pathways (Fig. 1B). The levels of ethanolamine and choline plasmalogens (PE’ and PC’, respectively), which in most cells account for ∼20% of membrane phospholipids, remained unchanged following LXR activation (Fig. 1C, D). In contrast, the level of the alkenyl lyso-PE and -PC species (LPE’ and LPC’, respectively) were markedly reduced by LXR activation, while the corresponding alkyl species (1-O-alkyl lysophosphatidylethanolamine and 1-O-alkyl lysophosphatidylcholine) were unchanged (Figs. 1D and S1B). Collectively, these results indicate that LXR stimulation reshapes the cellular lipid landscape and specifically decreases the level of lysoplasmalogens in macrophages.

**Fig. 2:**
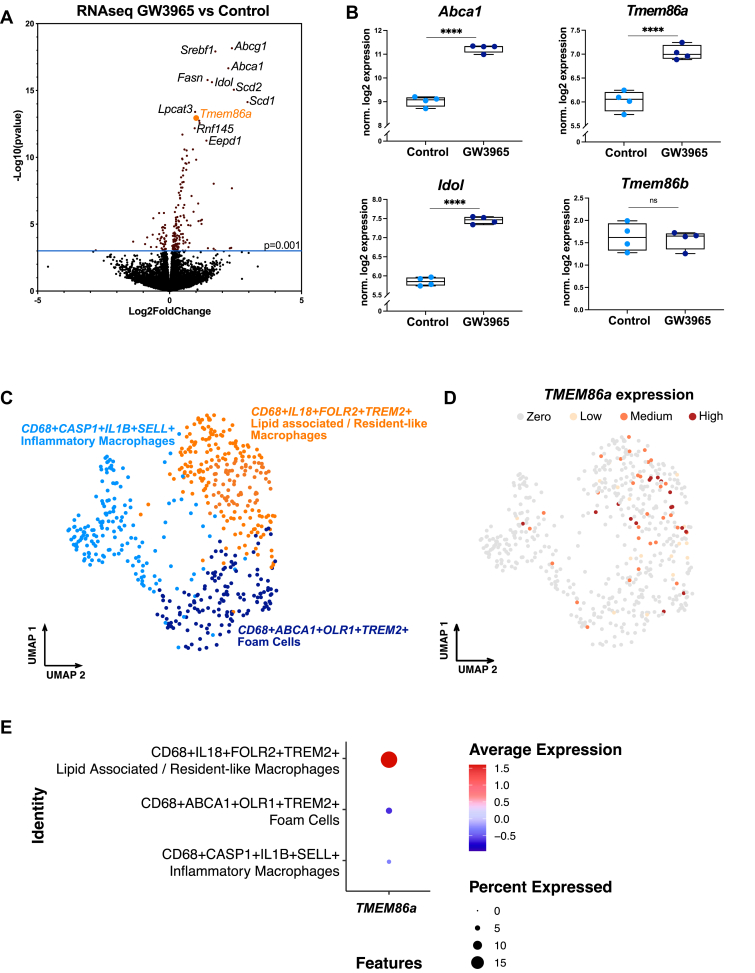
*TMEM86a* expression is increased by LXR activation and is associated with lipid accumulation in human plaque macrophages. *A*, *B*: Volcano plot of differential gene expression from BMDM (N = 4/group) stimulated with 1 μM GW3965 or vehicle for 6 h and corresponding results for *Abca1*, *Idol*, *Tmem86a,* and *Tmem86b*. C: scRNAseq of carotid plaques from 46 endarterectomy patients was performed, and macrophage clusters were identified. D: *TMEM86a* expression in individual cells is indicated as *Zero*: no *TMEM86a* expression. *Low*: bottom 25th percentile. *Medium*: between 25th and 75th percentile. *High*: Over 75th percentile of *TMEM86a* expression. E: *TMEM86a* expression is indicated in each macrophage cluster. ∗∗∗∗ *P*<0.0001.

As the level of PE’ and PC’ were unchanged after LXR activation, we reasoned that LXR may govern the production, or alternatively breakdown of the lysoplasmalogen derivatives. To identify the molecular mechanism underpinning the decrease in lysoplasmalogens we used RNAseq to map the global transcriptional response to LXR activation in BMDM. This resulted in the identification of 162 significantly up- and 31 significantly down-regulated genes. As anticipated, the expression of several bona fide LXR targets genes, including *Abca1* (3), *Idol* (5), *Rnf145* (58), *Lpcat3* (13, 14), and *Srebf1* (8) were increased (Fig. 2A, B and supplementary Table S6). Next to these established targets, we also observed that transmembrane protein 86a (*Tmem86a*) was increased by treatment with GW3965 (Fig. 2A, B). To obtain further support for the relevance of *TMEM86a* expression in macrophages to human disease we evaluated its expression in human atherosclerotic plaques. scRNA-seq on carotid plaques from 46 endarterectomy patients revealed an enrichment of *TMEM86a*-expressing cells in the lipid associated / resident-like macrophage population (Fig. 2C–E). Moreover, binning of *TMEM86a* expression in plaque macrophages showed a clear correlation of *TMEM86a* expression with the expression of lipid-related genes (supplemental Fig. S2). Hence, *Tmem86a* is expressed in murine and human macrophages and its regulation is associated with lipid accumulation-induced LXR signaling.

**Fig. 3:**
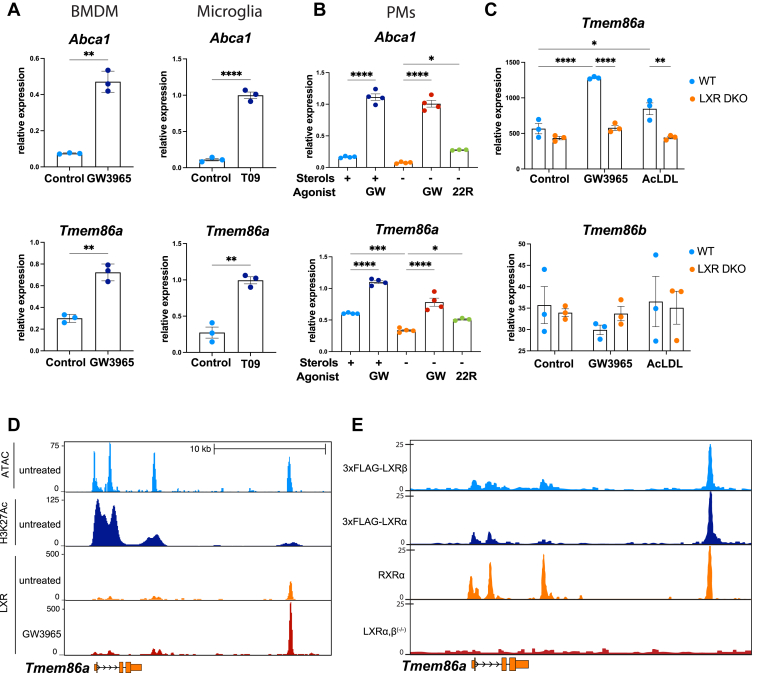
*Tmem86a* is a transcriptional target of LXR in macrophages and microglia. A: Cells were treated with the LXR agonists 1 μM GW3965 for 6 h or 1 μM T09 for 24 h. B: Cells were cultured for 16 h in the presence (+) or absence (-) of sterols before stimulation with 1 μM GW3965 or 5 μM *22R*-hydroxycholesterol. A, B: Expression of *Abca1* and *Tmem86a* was measured by qPCR in (A) BMDM and microglia or in (B) thioglycolate-elicited peritoneal macrophages (PMs). *Note:* Expression of *Tmem86b* was undetectable in these experiments. C: *Meta*-analysis of *Tmem86a* and *Tmem86b* expression in LXR wild-type and KO cells in GSE118656. D: ATAC-seq, H3K27Aac ChIP, and LXR ChIP-seq of the *Tmem86a* locus in (un)treated BMDM. The datasets GSE109965 and GSE79423 were analyzed. E: ChIP-seq (FLAG) was conducted on immortalized LXRα/β^(-/-)^ BMDM macrophages or those stably overexpressing flag-tagged LXRα or LXRβ. The RXRα lane was extracted from the sequence read archive (SRR1514119). Corresponding traces for the *Tmem86a* locus are shown. Mean ± SEM are depicted. ∗ *P*<0.05, ∗∗ *P*<0.01, ∗∗∗ *P*<0.001, ∗∗∗∗ *P*< 0.0001.

As a first step, we confirmed that expression of *Tmem86a,* but not of the closely related gene *Tmem86b* (59)*,* was increased ∼3-fold by pharmacological LXR activation in various macrophage subtypes; BMDM, thioglycolate-elicited PMs, and in murine microglia (Fig. 3A, B). Moreover, RNAseq analysis of LXR double-knockout macrophages revealed that regulation of *Tmem86a* was LXR-dependent in BMDMs and not inducible in the absence of LXR (Fig. 3C). Similar to pharmacologic activation by the synthetic ligands GW and T0901317, physiological-relevant inducers of LXR signaling in macrophages, such as desmosterol and AcLDL loading and a prototypic oxysterol, 22R-hydroxycholesterol, also increased *Tmem86a* expression (Figs. 3B, C and S3A–D). Intriguingly, expression of *Lpcat3* was induced by the synthetic LXR agonists but not by AcLDL in BMDM (supplemental Fig. S3A, B). Reciprocally, in line with LXR-dependent regulation of *Tmem86a,* sterol depletion reduced its expression (Fig. 3C), and stimulation with the proinflammatory bacterial cell-wall component lipopolysaccharide (LPS) markedly decreased its expression (supplemental Fig. S3C), congruent with previously reported downregulation of LXR target genes by LPS (60). We also evaluated whether *TMEM86a* is subject to regulation by LXR in human monocyte-derived macrophages. While *meta*-analysis of the LXR response of human PBMC showed clear upregulation of *TMEM86a* in response to an LXR ligand, our own measurements of human monocyte-derived macrophages treated with the LXR ligands T0901317 and GW were more variable and donor-dependent (supplemental Fig. S3E, F and data not shown). This may reflect differences in LXR levels between human and murine macrophages or some other yet-identified requirement for maximal *TMEM86a* induction in human cells. Finally, we interrogated the mode of *Tmem86a* regulation by LXRs. Congruent with direct regulation of *Tmem86a* by LXR, analysis of LXR genomic occupancy pointed toward a prominent ligand-dependent binding of both LXRα and LXRβ at an enhancer region located downstream of the *Tmem86a* locus (Fig. 3D, E). As such, our results point toward *Tmem86a* being a direct transcriptional target of LXRs in macrophages.

**Fig. 4:**
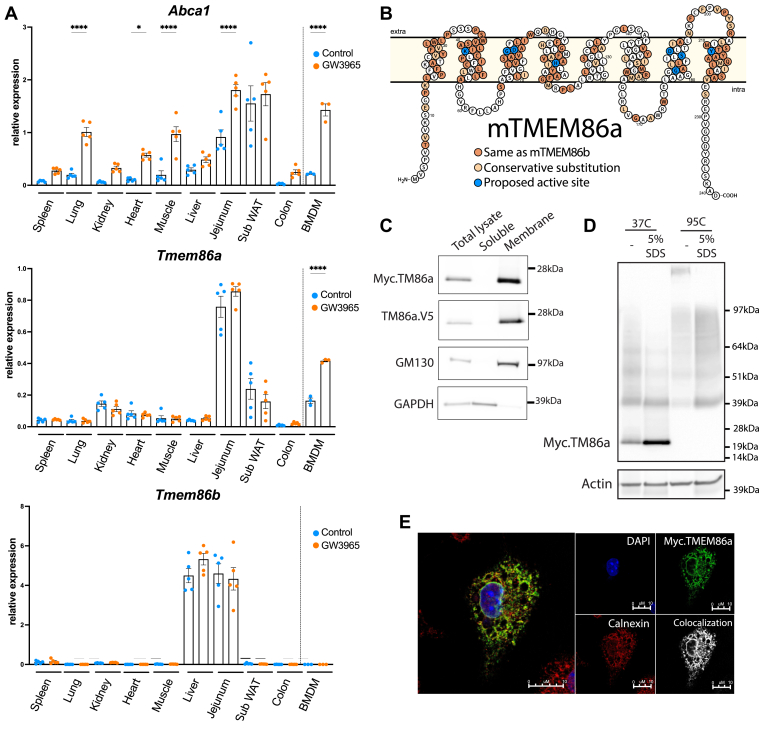
Tissue distribution and intracellular localization of TMEM86a. A: Wild-type mice (N = 5/group) were pharmacologically dosed with GW3965 for 4.5 days (20 mg/kg, twice daily). Organs were collected and gene expression determined by qPCR. BMDMs were isolated independently. B: Predicted membrane topology of mouse TMEM86a. Conservation with TMEM86b and residues predicted to form the catalytic site are indicated. C, D: Total cell lysates from HEK293T cells expressing Myc-TMEM86a or TMEM86a-V5 were fractionated and immunoblotted as indicated. GM130 and GAPDH were used to distinguish the membrane and cytosolic fractions, respectively. E: BMDMs were transduced with lentivirus encoding Myc-TMEM86a and stained as indicated. DAPI was used to counterstain the nuclei and cells were imaged by confocal microscopy. The mean ± SEM are depicted. ∗*P*<0.05, ∗∗∗∗*P* < 0.0001.

To extend our finding to the in vivo setting we determined the tissue distribution and LXR-dependent regulation of *Tmem86a* in WT mice dosed with GW. Expression of *Tmem86a* was markedly different from that of its homolog *Tmem86b* and was particularly high in jejunum, white adipose tissue, kidney, and macrophages (Fig. 4A). Distinct from macrophages, LXR activation in vivo*,* echoed by the induction of *Abca1* expression*,* failed to increase *Tmem86a* expression in these tissues. Therefore, regulation of *Tmem86a* by LXR may be restricted and limited to myeloid cells. *Tmem86a* encodes a 26 kDa membrane protein that belongs to the conserved family of bacterial YhhN proteins and is predicted to contain 8 transmembrane spanning domains (Fig. 4B). The YhhN family is highly conserved between species (59, 61), and accordingly TMEM86a has a high degree of sequence conservation with the only other known mammalian lysoplasmalogenase protein, TMEM86b (59). In accordance with the predicted membrane topology, TMEM86a protein was retrieved in the membrane fraction (Fig. 4C) and tended to form high molecular-weight aggregates that were SDS- and temperature-sensitive (Fig. 4D). Unfortunately, commercially available anti-TMEM86a antibodies failed to detect the endogenous mouse protein in macrophages precluding determining its native cellular localization. As an alternative, we used TMEM86a constructs containing either Myc or V5 tags on both the N and the C termini of the protein and determined the cellular localization of the heterologous protein. In BMDM and U2OS cells, TMEM86a colocalized with calnexin to the ER (Fig. 4E and data not shown). In BMDM, but not in U2OS cells, placing the affinity tag on the C terminus resulted in some redistribution of TMEM86a to the Golgi where it colocalized with Golgin (data not show). This may suggest that in BMDM, the cytoplasm-facing C-terminal tail may be a determinant of ER localization of TMEM86a.

**Fig. 5:**
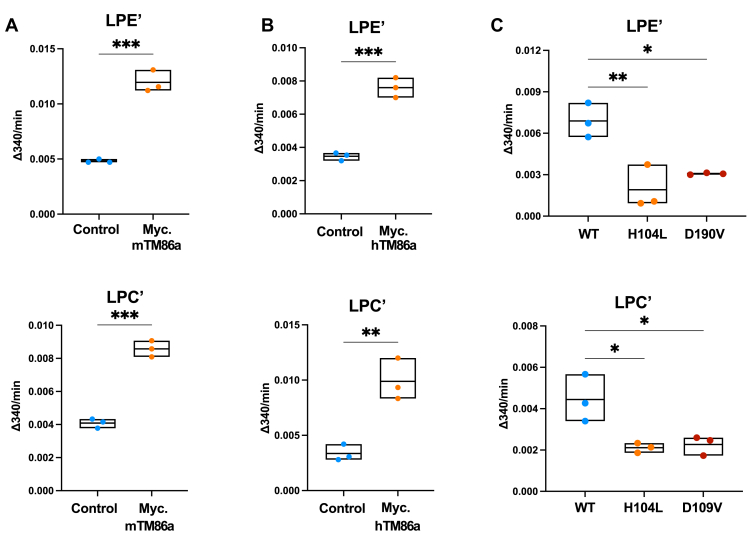
TMEM86a has lysoplasmalogenase activity. *A*, *B*: HEK293T cells were transfected with the indicated mouse or human TMEM86a constructs. Crude membrane fractions were isolated, and the rate of LPE’ and LPC’ hydrolysis was determined biochemically in vitro. C: The indicated mutations were introduced in murine TMEM86a and LPE’ and LPC’ hydrolysis determined as above. The mean ± SEM change in absorbance at 340 nM from three independent experiments is plotted. ∗ *P* <0.05, ∗∗ *P* <0.01, ∗∗∗ *P* <0.001.

TMEM86a is related to TMEM86b and other bacterial YhhN proteins (Fig. 4B), which were reported to have lysoplasmalogenase activity (59, 62), and a recent report described TMEM86a as a lysoplasmalogenase in adipose tissue (63). We therefore reasoned that TMEM86a may have similar activity in macrophages as well, which could explain the effect of LXR activation on LPE’ and LPC’ levels in these cells. To directly test this notion, we adapted a biochemical assay that couples the release of the lysoplasmalogen *sn-1* fatty aldehyde to oxidation of NADH by ADH (supplemental Fig. S4A). In line with the lipidomic analysis in macrophages (Fig. 1), we found that microsomal fractions from HEK293T cells overproducing mTMEM86a or hTMEM86a had a ∼2–2.5-fold increase in the hydrolysis rate of both LPC’ and LPE’ (Figs. 5A, B and S4B, C). To further characterize the enzymatic activity, we introduced five mutations predicted to disrupt the catalytic site according to homology with other YhhN family members (61, 62). Of these, only two produced protein at comparable levels to the wild-type protein and were studied further (supplemental Fig. S4D, E), and consistent with having a catalytic role, mutations p.H104L and p.D190V resulted in a decrease in lysoplasmalogenase activity (Fig. 5C). These experiments support the contention that both mouse and human TMEM86a have lysoplasmalogenase activity in a reconstituted biochemical assay.

**Fig. 6:**
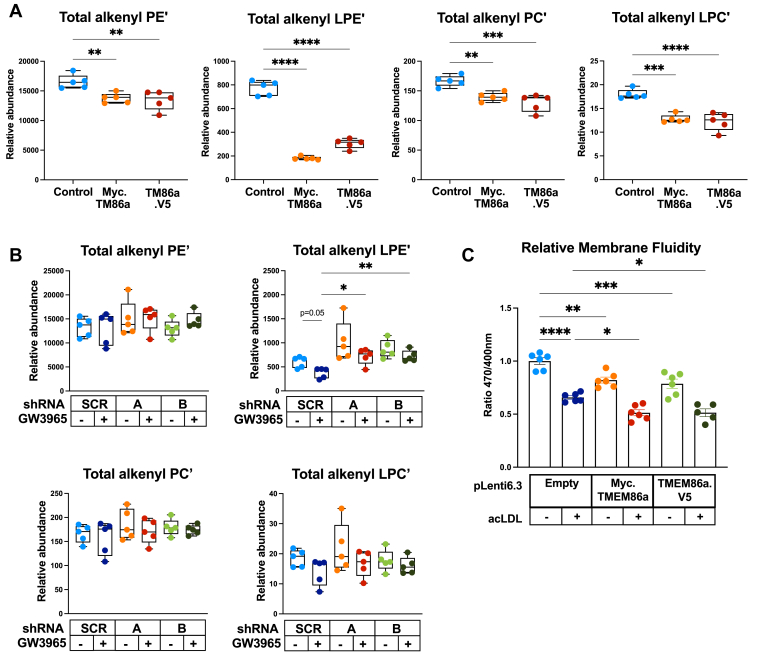
Endogenous lysoplasmalogen levels are sensitive to TMEM86a expression. A, B: Lipidomic analysis of the indicated lipid species following (A, C) overexpression, or (B) shRNA-mediated silencing of TMEM86a in BMDM (N = 5/group). Where indicated cells were also stimulated with 1 μM GW3965 for 16 h. C: Cells were cultured with 50 μg/ml AcLDL for 16 h as indicated after which membrane fluidity was measured (N = 6). The mean ± SEM are plotted. ∗ *P*<0.05, ∗∗ *P* <0.01, ∗∗∗ *P* <0.001*,* ∗∗∗∗ *P* < 0.0001.

To evaluate whether this activity is present in a cellular setting, we either overexpressed or silenced *Tmem86a* in primary mouse BMDMs using an optimized lentiviral transduction approach (supplemental Fig. S5A). Overexpressed N- or C-terminally tagged TMEM86a was readily detectable in transduced BMDM (supplemental Fig. S5B) and resulted in large reduction of cellular LPE’ and LPC’, independent of acyl chain length or saturation (Fig. 6A and supplemental Table S7). Of note, this treatment had no effect on the level of other LXR- and SREBP-regulated target genes (supplemental Fig. S5C). We point out that in this overexpression setting, we also observed a reduction of the parent PE’ and PC’ species, which was not observed following pharmacological LXR activation (Fig. 1D). This may reflect the elevated *Tmem86a* levels in this setting as compared to that attained following LXR activation, which may limit regeneration of plasmalogens via reacylation of the generated lysoplasmalogen (supplemental Fig. S5C). Reciprocally, to evaluate the consequence of *Tmem86a* silencing, we selected two independent *Tmem86a-*targeting shRNAs based on their ability to silence cotransfected *Tmem86a* in HEK293T cells and to decrease basal and LXR-stimulated expression of *Tmem86a* in BMDM (supplemental Fig. S6A ,B). We initially evaluated the involvement of *Tmem86a* in several well-established LXR-regulated processes and found that effective shRNA-mediated silencing of *Tmem86a* did not change overall LXR and SREBP signaling (supplemental Fig. S6C, D). Similarly, gain or loss of *Tmem86a* expression did not alter LPS-induced inflammatory gene expression (supplemental Fig. S7A, B), the transcriptional response to AcLDL uptake (i.e., increased LXR and decreased SREBP signaling, respectively, supplemental Fig. S7C), zymosan phagocytosis or LXR-stimulated cholesterol efflux to ApoAI and HDL (data not shown). However, consistent with the overexpression studies, *Tmem86a* silencing led to an increase in the basal level of LPE’, the most prominent lysoplasmalogen species in BMDM, and largely attenuated the ability of LXR activation to reduce this lipid species (Fig. 6B and supplemental Table S7). LPC’ levels, which are substantially lower than those of LPE’ in BMDM (∼30-fold lower), remained unchanged in this experimental setting. Since lysoplasmalogens are known to influence membrane fluidity, we evaluated the role of TMEM86a herein. We found that TMEM86a overexpression resulted in reduced membrane fluidity, which was additive to the decrease induced by AcLDL (Fig. 6C). Silencing of *Tmem86a* did not modify membrane fluidity in this setting, possibly reflecting the more subtle effect this has on membrane composition (data not shown). In aggregate, these loss-of-function and gain-of-function studies position TMEM86a as a sterol-inducible lysoplasmalogenase that remodels the membrane composition in macrophages.

## DISCUSSION

The regulation of membrane composition is a fundamental process that is dynamically regulated in order to adapt to a wide range of diverse metabolic cues and signaling pathways. Plasmalogens are a major constituent of cellular membranes, yet the pathways that govern their turnover are not well understood (19, 20). Our study highlights the critical contribution of LXR-regulated signaling to this process in macrophages. As such, our key finding is the identification of *TMEM86a* as a sterol-inducible lysoplasmalogenase that directly governs lysoplasmalogen catabolism in macrophages.

Until recently, TMEM86b was the sole characterized mammalian lysoplasmalogenase (59). During the course of our study, Cho *et al.* described the generation of adipose-specific *Tmem86a* knockout mice and demonstrated that it has lysoplasmalogenase activity in this tissue (63). We further extend the role of TMEM86a as a sterol-regulated lysoplasmalogenase in macrophages. Collectively these studies position TMEM86a as the second mammalian lysoplasmalogenase next to TMEM86b. While the catalytic activity and substrate specificity of TMEM86a/b are similar, their tissue distribution and sterol-dependent induction clearly demarcates them. Consistent with its tissue distribution, high TMEM86b-associated activity has been reported to mediate the catabolism of lysoplasmalogens in liver-derived microsomes (57, 59), which may, at least in part, contribute to the low abundance of this lipid species in the liver. Accordingly, Wu *et al.* proposed that in the liver, TMEM86b acts to attenuate the potential membrane-disrupting properties of lysoplasmalogens carried on or generated following the endocytosis of LDL (59). In contrast to *Tmem86b*, we do not detect *Tmem86a* expression in primary hepatocytes or in immortalized hepatocyte-like cell lines. Instead, *Tmem86a* is abundant in myeloid cells where its expression is subject to LXR-dependent regulation, suggesting that its associated activity is coupled to increased levels of cellular sterols. Circulating plasma lipoproteins are rich in plasmalogens (64) and also carry small amounts of lysoplasmalogens (personal communication, dr. J. Kroon, Academic Medical Center, University of Amsterdam). A reigning notion is that the plasmalogens act to protect lipoproteins, amongst others, from oxidation (64, 65) and that increasing plasmalogens on lipoproteins can attenuate atherosclerosis in ApoE-null mice (66). As uptake of modified lipoproteins by macrophages increases cellular plasmalogen levels (67), it follows that in order to maintain membrane integrity, these need to be broken down in a process that inevitably will also generate lysoplasmalogens. In this setting, LXR-dependent induction of *Tmem86a* will limit reacylation of lysoplasmalogens and may prevent accumulation of potentially membrane-disrupting lysoplasmalogen species (68, 69). We demonstrate that in the absence of *Tmem86a,* LXR activation fails to reduce lysoplasmalogen levels in macrophages, and hence, we speculate that TMEM86a may act on lipoprotein-derived (lyso)plasmalogens.

Our study also raises the intriguing question as to why LXR simultaneously regulates both LPCAT3 and TMEM86a, which at face value may seem contradictory. LPCAT3 preferentially transfers a PUFA from acyl-CoA to LPE’ and LPC’ (13, 16, 18), albeit with lower efficiency than to LPC and LPE, ultimately resulting in increased desaturation of plasmalogens in macrophages (27). This change in saturation was not associated with a difference in the absolute abundance of plasmalogens when *Lpcat3* was absent in macrophages (27). The levels of lysoplasmalogens were not reported in this study. Similar to LPCAT3, loss of *Tmem86a* expression does not alter plasmalogens levels in BMDM yet increases the level of LPE’ and LPC’. Moreover, as we report the ability of LXR to reduce lysoplasmalogen levels in the absence of *Tmem86a* is largely attenuated*,* also suggesting that in this setting LPCAT3-mediated reacylation of this lipid species is less prominent. Intriguingly, TMEM86b activity has been inversely correlated with cellular plasmalogen content (59), which is in line with our observation that overexpression of *Tmem86a* leads to a concomitant decrease in plasmalogens and lysoplasmalogens. Maintaining plasmalogen homeostasis by preventing lysoplasmalogen reacetylation may therefore be an important facet of TMEM86a function. Another notable difference between TMEM86a and LPCAT3 is that the latter is activated as part of an anabolic LXR-regulated pathway that promotes both fatty acid and cholesterol biosynthesis (16, 70, 71). TMEM86a on the other hand is part of a catabolic process, and our results do not support a role in the aforementioned processes as both gain or loss of its functions did not alter the SREBP1 or SREBP2 signaling pathways at the gene expression or protein levels. Additionally, supporting our hypothesis on the role of TMEM86a in processing lipoprotein-derived (lyso)plasmalogens, we found that *Tmem86a* expression is increased by AcLDL loading of macrophages, but that of *Lpcat3* is refractory to this treatment. It is therefore possible the divergent roles of TMEM86a and LPCAT3 in LXR-regulated membrane remodeling may be context and cell-type dependent.

Plasmalogens have been linked to cholesterol metabolism through their ability to modulate the transport (72, 73) and biosynthesis of cholesterol (74), next to their effects on the biophysical properties of membranes (19). Whether lysoplasmalogens have a regulatory role in cholesterol metabolism in macrophages or are they merely a breakdown metabolite of plasmalogens is unknown at present. We point out that the majority of studies looking at the physiological functions of plasmalogens rely on the use of plasmalogen biosynthesis-impaired cells and therefore do not discriminate between the functions of plasmalogens and lysoplasmalogens. We have evaluated several established LXR-regulated processes in macrophages, but these were independent of *Tmem86a* expression. A limitation of these studies is their reliance on shRNA-mediated silencing of *Tmem86a* expression in primary macrophages, which may leave residual TMEM86a activity. The recently reported loss-of-function *Tmem86a* mice will greatly facilitate uncovering the physiological role of TMEM86a-mediated membrane remodeling (63). In conclusion, our study highlights the increasingly complex role LXR have in governing membrane remodeling in macrophages and emphasizes the need to uncover the function(s) of TMEM86a and lysoplasmalogens in these cells in health and disease.

## Data availability

The datasets generated during and/or analyzed during the current study are available as supplementary material. Reagents used in the study are available from the corresponding author on reasonable request.

## Supplemental data

This article contains supplemental data.

## Conflict of interest

The authors declare no conflict of interest.

## References

[1] Janowski B.A., Willy P.J., Devi T.R., Falck J.R., Mangelsdorf D.J. (1996). An oxysterol signalling pathway mediated by the nuclear receptor LXR alpha. Nature.

[2] Yang C., McDonald J.G., Patel A., Zhang Y., Umetani M., Xu F. (2006). Sterol intermediates from cholesterol biosynthetic pathway as liver X receptor ligands. J. Biol. Chem..

[3] Venkateswaran A., Laffitte B.A., Joseph S.B., Mak P.A., Wilpitz D.C., Edwards P.A. (2000). Control of cellular cholesterol efflux by the nuclear oxysterol receptor LXR alpha. Proc. Natl. Acad. Sci. U. S. A..

[4] Kennedy M.A., Barrera G.C., Nakamura K., Baldan A., Tarr P., Fishbein M.C. (2005). ABCG1 has a critical role in mediating cholesterol efflux to HDL and preventing cellular lipid accumulation. Cell Metab..

[5] Zelcer N., Hong C., Boyadjian R., Tontonoz P. (2009). LXR regulates cholesterol uptake through Idol-dependent ubiquitination of the LDL receptor. Science.

[6] Sorrentino V., Zelcer N. (2012). Post-transcriptional regulation of lipoprotein receptors by the E3-ubiquitin ligase inducible degrader of the low-density lipoprotein receptor. Curr. Opin. Lipidol..

[7] Schultz J.R., Tu H., Luk A., Repa J.J., Medina J.C., Li L. (2000). Role of LXRs in control of lipogenesis. Genes Dev..

[8] Repa J.J., Liang G., Ou J., Bashmakov Y., Lobaccaro J.M., Shimomura I. (2000). Regulation of mouse sterol regulatory element-binding protein-1c gene (SREBP-1c) by oxysterol receptors, LXRalpha and LXRbeta. Genes Dev..

[9] Zelcer N., Tontonoz P. (2006). Liver X receptors as integrators of metabolic and inflammatory signaling. J. Clin. Invest..

[10] Hong C., Tontonoz P. (2014). Liver X receptors in lipid metabolism: opportunities for drug discovery. Nat. Rev. Drug Discov..

[11] Wang B., Tontonoz P. (2018). Liver X receptors in lipid signalling and membrane homeostasis. Nat. Rev. Endocrinol..

[12] Zelcer N., Khanlou N., Clare R., Jiang Q., Reed-Geaghan E.G., Landreth G.E. (2007). Attenuation of neuroinflammation and Alzheimer's disease pathology by liver x receptors. Proc. Natl. Acad. Sci. U. S. A..

[13] Rong X., Albert C.J., Hong C., Duerr M.A., Chamberlain B.T., Tarling E.J. (2013). LXRs regulate ER stress and inflammation through dynamic modulation of membrane phospholipid composition. Cell Metab..

[14] Demeure O., Lecerf F., Duby C., Desert C., Ducheix S., Guillou H. (2011). Regulation of LPCAT3 by LXR. Gene.

[15] Lagrost L., Masson D. (2022). The expanding role of lyso-phosphatidylcholine acyltransferase-3 (LPCAT3), a phospholipid remodeling enzyme, in health and disease. Curr. Opin. Lipidol..

[16] Rong X., Wang B., Dunham M.M., Hedde P.N., Wong J.S., Gratton E. (2015). Lpcat3-dependent production of arachidonoyl phospholipids is a key determinant of triglyceride secretion. Elife.

[17] Gijon M.A., Riekhof W.R., Zarini S., Murphy R.C., Voelker D.R. (2008). Lysophospholipid acyltransferases and arachidonate recycling in human neutrophils. J. Biol. Chem..

[18] Zhao Y., Chen Y.Q., Bonacci T.M., Bredt D.S., Li S., Bensch W.R. (2008). Identification and characterization of a major liver lysophosphatidylcholine acyltransferase. J. Biol. Chem..

[19] Jimenez-Rojo N., Riezman H. (2019). On the road to unraveling the molecular functions of ether lipids. FEBS Lett..

[20] Braverman N.E., Moser A.B. (2012). Functions of plasmalogen lipids in health and disease. Biochim. Biophys. Acta.

[21] Lohner K., Balgavy P., Hermetter A., Paltauf F., Laggner P. (1991). Stabilization of non-bilayer structures by the etherlipid ethanolamine plasmalogen. Biochim. Biophys. Acta.

[22] Dorninger F., Forss-Petter S., Wimmer I., Berger J. (2020). Plasmalogens, platelet-activating factor and beyond - Ether lipids in signaling and neurodegeneration. Neurobiol. Dis..

[23] Brosche T., Platt D. (1998). The biological significance of plasmalogens in defense against oxidative damage. Exp. Gerontol..

[24] Zoeller R.A., Lake A.C., Nagan N., Gaposchkin D.P., Legner M.A., Lieberthal W. (1999). Plasmalogens as endogenous antioxidants: somatic cell mutants reveal the importance of the vinyl ether. Biochem. J..

[25] Gil-de-Gomez L., Astudillo A.M., Lebrero P., Balboa M.A., Balsinde J. (2017). Essential role for ethanolamine plasmalogen hydrolysis in bacterial lipopolysaccharide priming of macrophages for enhanced arachidonic acid release. Front. Immunol..

[26] Menegaut L., Jalil A., Thomas C., Masson D. (2019). Macrophage fatty acid metabolism and atherosclerosis: the rise of PUFAs. Atherosclerosis.

[27] Thomas C., Jalil A., Magnani C., Ishibashi M., Quere R., Bourgeois T. (2018). LPCAT3 deficiency in hematopoietic cells alters cholesterol and phospholipid homeostasis and promotes atherosclerosis. Atherosclerosis.

[28] Paul S., Lancaster G.I., Meikle P.J. (2019). Plasmalogens: a potential therapeutic target for neurodegenerative and cardiometabolic disease. Prog. Lipid Res..

[29] Nelson J.K., Koenis D.S., Scheij S., Cook E.C., Moeton M., Santos A. (2017). EEPD1 Is a Novel LXR Target Gene in Macrophages Which Regulates ABCA1 Abundance and Cholesterol Efflux. Arterioscler. Thromb. Vasc. Biol..

[30] Grajchen E., Wouters E., van de Haterd B., Haidar M., Hardonniere K., Dierckx T. (2020). CD36-mediated uptake of myelin debris by macrophages and microglia reduces neuroinflammation. J. Neuroinflam..

[31] Jorissen W., Wouters E., Bogie J.F., Vanmierlo T., Noben J.P., Sviridov D. (2017). Relapsing-remitting multiple sclerosis patients display an altered lipoprotein profile with dysfunctional HDL. Sci. Rep..

[32] Vert J.P., Foveau N., Lajaunie C., Vandenbrouck Y. (2006). An accurate and interpretable model for siRNA efficacy prediction. BMC Bioinform..

[33] Tan J.M.E., van der Stoel M.M., van den Berg M., van Loon N.M., Moeton M., Scholl E. (2020). The MARCH6-SQLE Axis Controls Endothelial Cholesterol Homeostasis and Angiogenic Sprouting. Cell Rep..

[34] Tsirigos K.D., Peters C., Shu N., Kall L., Elofsson A. (2015). The TOPCONS web server for consensus prediction of membrane protein topology and signal peptides. Nucl. Acids Res..

[35] Vaz F.M., McDermott J.H., Alders M., Wortmann S.B., Kolker S., Pras-Raves M.L. (2019). Mutations in PCYT2 disrupt etherlipid biosynthesis and cause a complex hereditary spastic paraplegia. Brain.

[36] Clemente-Olivo M.P., Habibe J.J., Vos M., Ottenhoff R., Jongejan A., Herrema H. (2021). Four-and-a-half LIM domain protein 2 (FHL2) deficiency protects mice from diet-induced obesity and high FHL2 expression marks human obesity. Metabolism.

[37] Sayols S., Scherzinger D., Klein H. (2016). dupRadar: a Bioconductor package for the assessment of PCR artifacts in RNA-Seq data. BMC Bioinform..

[38] Bolger A.M., Lohse M., Usadel B. (2014). Trimmomatic: a flexible trimmer for Illumina sequence data. Bioinformatics.

[39] Kim D., Langmead B., Salzberg S.L. (2015). HISAT: a fast spliced aligner with low memory requirements. Nat. Met..

[40] Anders S., Pyl P.T., Huber W. (2015). HTSeq--a Python framework to work with high-throughput sequencing data. Bioinformatics.

[41] Robinson M.D., McCarthy D.J., Smyth G.K. (2010). edgeR: a Bioconductor package for differential expression analysis of digital gene expression data. Bioinformatics.

[42] Ritchie M.E., Phipson B., Wu D., Hu Y., Law C.W., Shi W. (2015). limma powers differential expression analyses for RNA-sequencing and microarray studies. Nucl. Acids Res..

[43] Hoeksema M.A., Shen Z., Holtman I.R., Zheng A., Spann N.J., Cobo I. (2021). Mechanisms underlying divergent responses of genetically distinct macrophages to IL-4. Sci. Adv..

[44] Oishi Y., Spann N.J., Link V.M., Muse E.D., Strid T., Edillor C. (2017). SREBP1 contributes to resolution of pro-inflammatory TLR4 signaling by reprogramming fatty acid metabolism. Cell Metab..

[45] Langmead B., Salzberg S.L. (2012). Fast gapped-read alignment with Bowtie 2. Nat. Met..

[46] Heinz S., Benner C., Spann N., Bertolino E., Lin Y.C., Laslo P. (2010). Simple combinations of lineage-determining transcription factors prime cis-regulatory elements required for macrophage and B cell identities. Mol. Cell.

[47] Ramon-Vazquez A., de la Rosa J.V., Tabraue C., Lopez F., Diaz-Chico B.N., Bosca L. (2019). Common and differential transcriptional actions of nuclear receptors liver X receptors alpha and beta in macrophages. Mol. Cell Biol..

[48] Daniel B., Nagy G., Hah N., Horvath A., Czimmerer Z., Poliska S. (2014). The active enhancer network operated by liganded RXR supports angiogenic activity in macrophages. Genes Dev..

[49] Liebergall S.R., Angdisen J., Chan S.H., Chang Y., Osborne T.F., Koeppel A.F. (2020). Inflammation triggers liver X receptor-dependent lipogenesis. Mol. Cell Biol..

[50] Muse E.D., Yu S., Edillor C.R., Tao J., Spann N.J., Troutman T.D. (2018). Cell-specific discrimination of desmosterol and desmosterol mimetics confers selective regulation of LXR and SREBP in macrophages. Proc. Natl. Acad. Sci. U. S. A..

[51] Pehkonen P., Welter-Stahl L., Diwo J., Ryynanen J., Wienecke-Baldacchino A., Heikkinen S. (2012). Genome-wide landscape of liver X receptor chromatin binding and gene regulation in human macrophages. BMC Genomics.

[52] Depuydt M.A.C., Prange K.H.M., Slenders L., Ord T., Elbersen D., Boltjes A. (2020). Microanatomy of the Human Atherosclerotic Plaque by Single-Cell Transcriptomics. Circ. Res..

[53] Hao Y., Hao S., Andersen-Nissen E., Mauck W.M., Zheng S., Butler A. (2021). Integrated analysis of multimodal single-cell data. Cell.

[54] Choudhary S., Satija R. (2022). Comparison and evaluation of statistical error models for scRNA-seq. Genome Biol..

[55] Wickham H. (2016).

[56] Loregger A., Raaben M., Nieuwenhuis J., Tan J.M.E., Jae L.T., van den Hengel L.G. (2020). Haploid genetic screens identify SPRING/C12ORF49 as a determinant of SREBP signaling and cholesterol metabolism. Nat. Commun..

[57] Jurkowitz-Alexander M., Ebata H., Mills J.S., Murphy E.J., Horrocks L.A. (1989). Solubilization, purification and characterization of lysoplasmalogen alkenylhydrolase (lysoplasmalogenase) from rat liver microsomes. Biochim. Biophys. Acta.

[58] Cook E.C., Nelson J.K., Sorrentino V., Koenis D., Moeton M., Scheij S. (2017). Identification of the ER-resident E3 ubiquitin ligase RNF145 as a novel LXR-regulated gene. PLoS One.

[59] Wu L.C., Pfeiffer D.R., Calhoon E.A., Madiai F., Marcucci G., Liu S. (2011). Purification, identification, and cloning of lysoplasmalogenase, the enzyme that catalyzes hydrolysis of the vinyl ether bond of lysoplasmalogen. J. Biol. Chem..

[60] Joseph S.B., Castrillo A., Laffitte B.A., Mangelsdorf D.J., Tontonoz P. (2003). Reciprocal regulation of inflammation and lipid metabolism by liver X receptors. Nat. Med..

[61] Ovchinnikov S., Kinch L., Park H., Liao Y., Pei J., Kim D.E. (2015). Large-scale determination of previously unsolved protein structures using evolutionary information. Elife.

[62] Jurkowitz M.S., Patel A., Wu L.C., Krautwater A., Pfeiffer D.R., Bell C.E. (2015). The YhhN protein of Legionella pneumophila is a Lysoplasmalogenase. Biochim. Biophys. Acta.

[63] Cho Y.K., Yoon Y.C., Im H., Son Y., Kim M., Saha A. (2022). Adipocyte lysoplasmalogenase TMEM86A regulates plasmalogen homeostasis and protein kinase A-dependent energy metabolism. Nat. Commun..

[64] Brautigam C., Engelmann B., Reiss D., Reinhardt U., Thiery J., Richter W.O. (1996). Plasmalogen phospholipids in plasma lipoproteins of normolipidemic donors and patients with hypercholesterolemia treated by LDL apheresis. Atherosclerosis.

[65] Hahnel D., Thiery J., Brosche T., Engelmann B. (1999). Role of plasmalogens in the enhanced resistance of LDL to copper-induced oxidation after LDL apheresis. Arterioscler. Thromb. Vasc. Biol..

[66] Rasmiena A.A., Barlow C.K., Stefanovic N., Huynh K., Tan R., Sharma A. (2015). Plasmalogen modulation attenuates atherosclerosis in ApoE- and ApoE/GPx1-deficient mice. Atherosclerosis.

[67] Wallner S., Orso E., Grandl M., Konovalova T., Liebisch G., Schmitz G. (2018). Phosphatidylcholine and phosphatidylethanolamine plasmalogens in lipid loaded human macrophages. PLoS One.

[68] Han X.L., Gross R.W. (1991). Alterations in membrane dynamics elicited by amphiphilic compounds are augmented in plasmenylcholine bilayers. Biochim. Biophys. Acta.

[69] Caldwell R.A., Baumgarten C.M. (1998). Plasmalogen-derived lysolipid induces a depolarizing cation current in rabbit ventricular myocytes. Circ. Res..

[70] Rong X., Wang B., Palladino E.N., de Aguiar Vallim T.Q., Ford D.A., Tontonoz P. (2017). ER phospholipid composition modulates lipogenesis during feeding and in obesity. J. Clin. Invest..

[71] Wang B., Rong X., Palladino E.N.D., Wang J., Fogelman A.M., Martin M.G. (2018). Phospholipid Remodeling and Cholesterol Availability Regulate Intestinal Stemness and Tumorigenesis. Cell Stem Cell.

[72] Maeba R., Kojima K.I., Nagura M., Komori A., Nishimukai M., Okazaki T. (2018). Association of cholesterol efflux capacity with plasmalogen levels of high-density lipoprotein: a cross-sectional study in chronic kidney disease patients. Atherosclerosis.

[73] Munn N.J., Arnio E., Liu D., Zoeller R.A., Liscum L. (2003). Deficiency in ethanolamine plasmalogen leads to altered cholesterol transport. J. Lipid Res..

[74] Honsho M., Abe Y., Fujiki Y. (2015). Dysregulation of Plasmalogen Homeostasis Impairs Cholesterol Biosynthesis. J. Biol. Chem..

