# Supplementary material for: Sterol-regulated transmembrane protein TMEM86a couples LXR signaling to regulation of lysoplasmalogens in macrophages
Source: J Lipid Res. 2022 Dec 31;64(2):100325. doi: 10.1016/j.jlr.2022.100325 (PMC9926310; doi:10.1016/j.jlr.2022.100325)

Supplemental figure 1

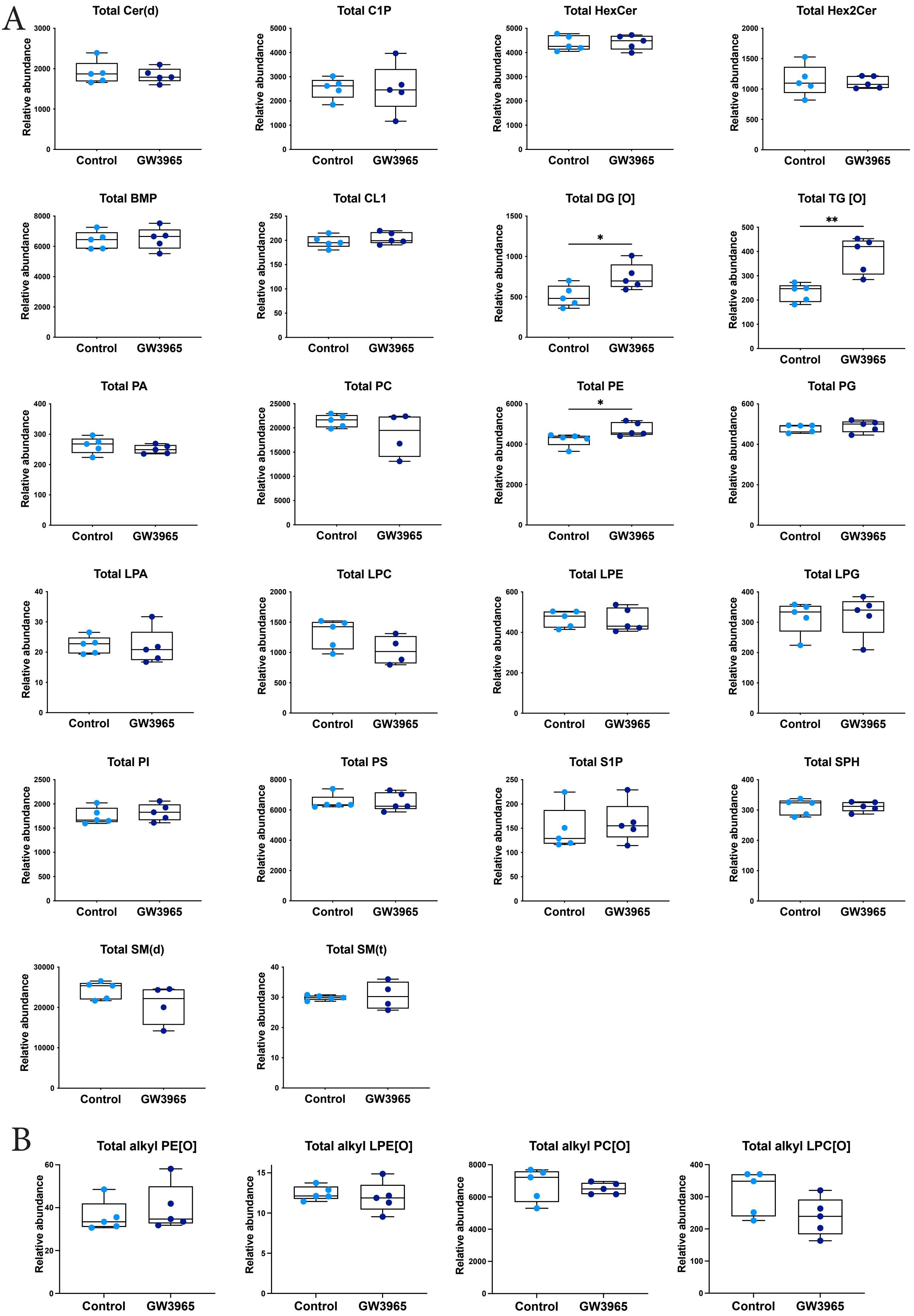

Supplemental figure 2

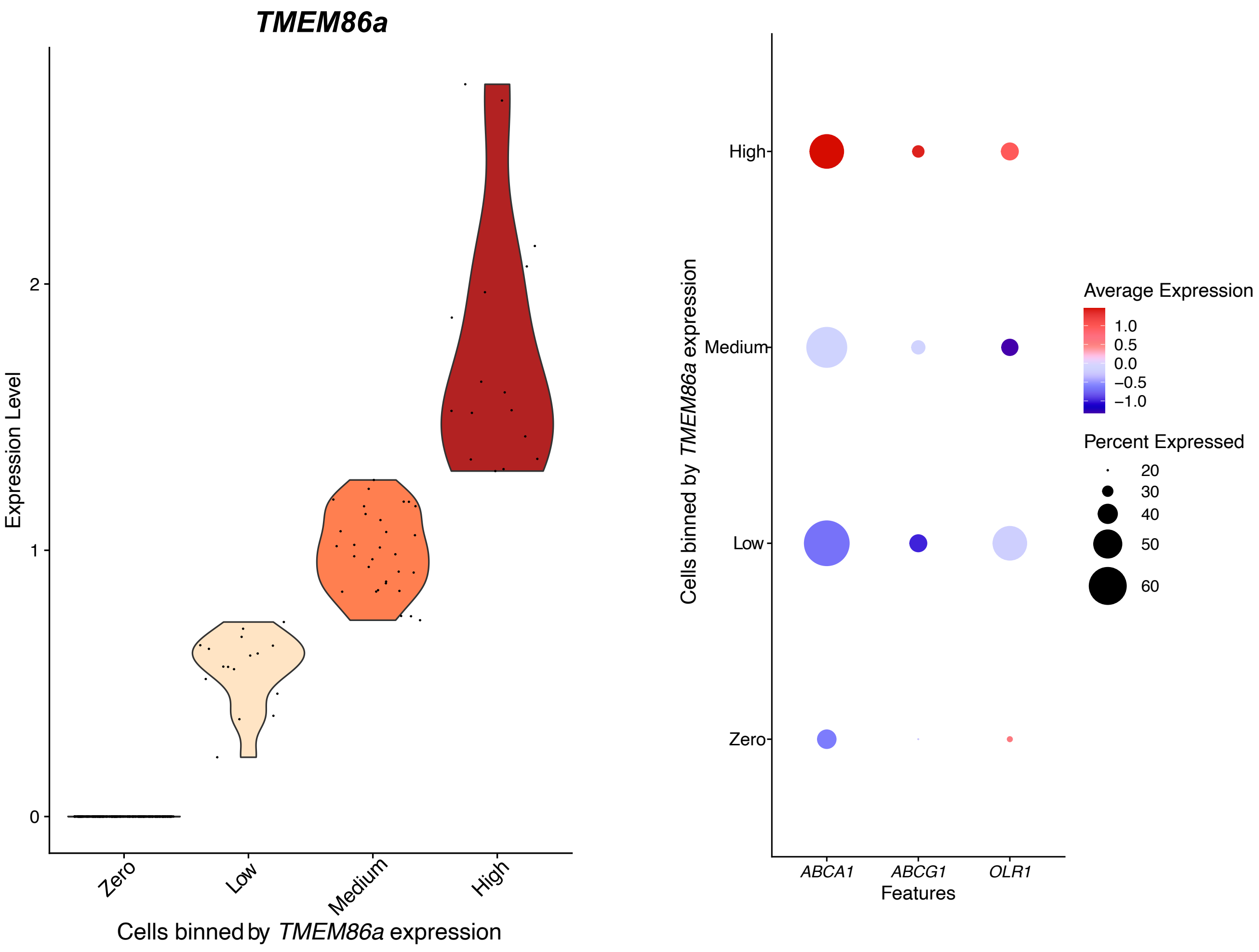

Supplemental figure 3

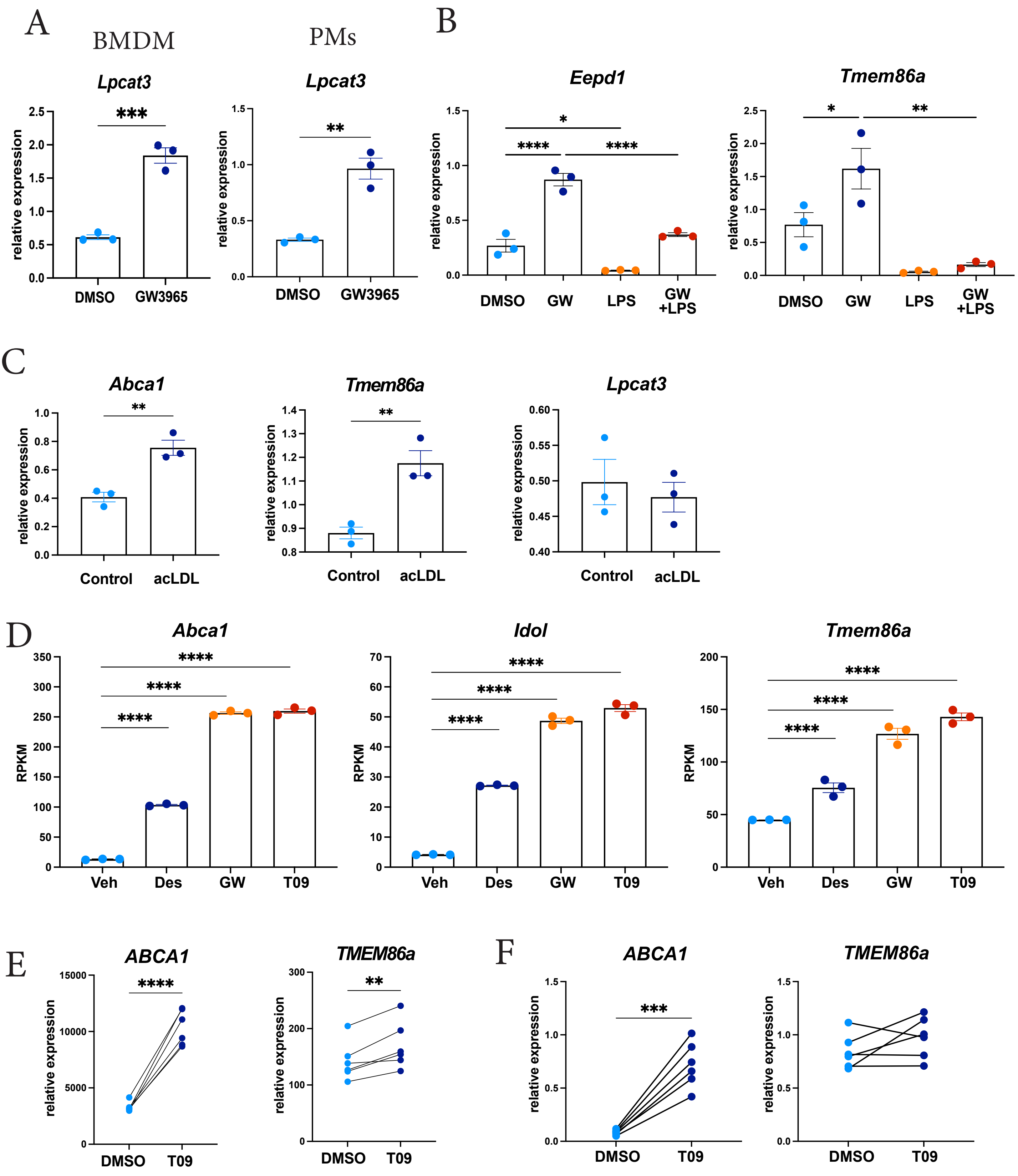

Supplemental figure 4

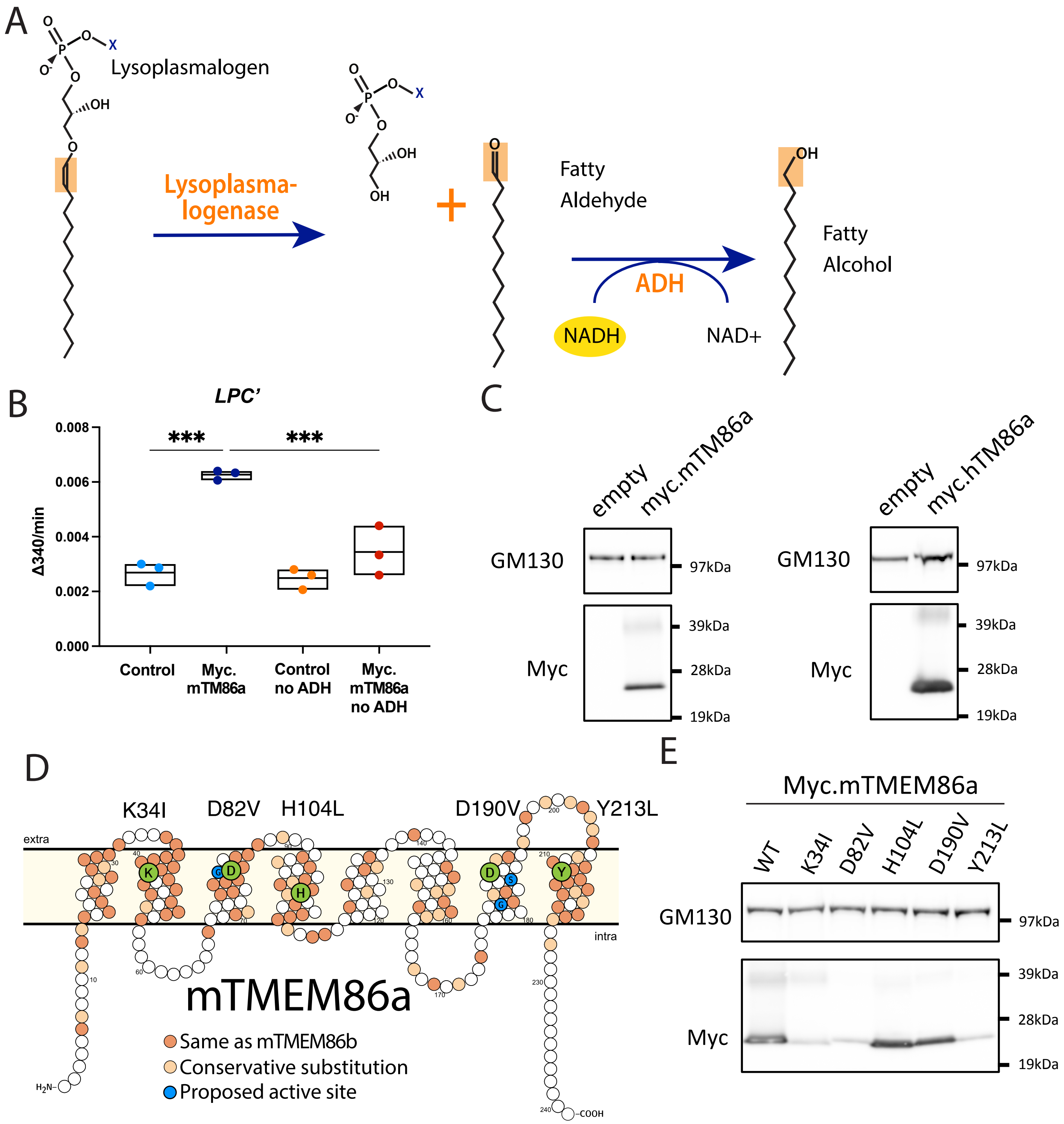

Supplemental figure 5

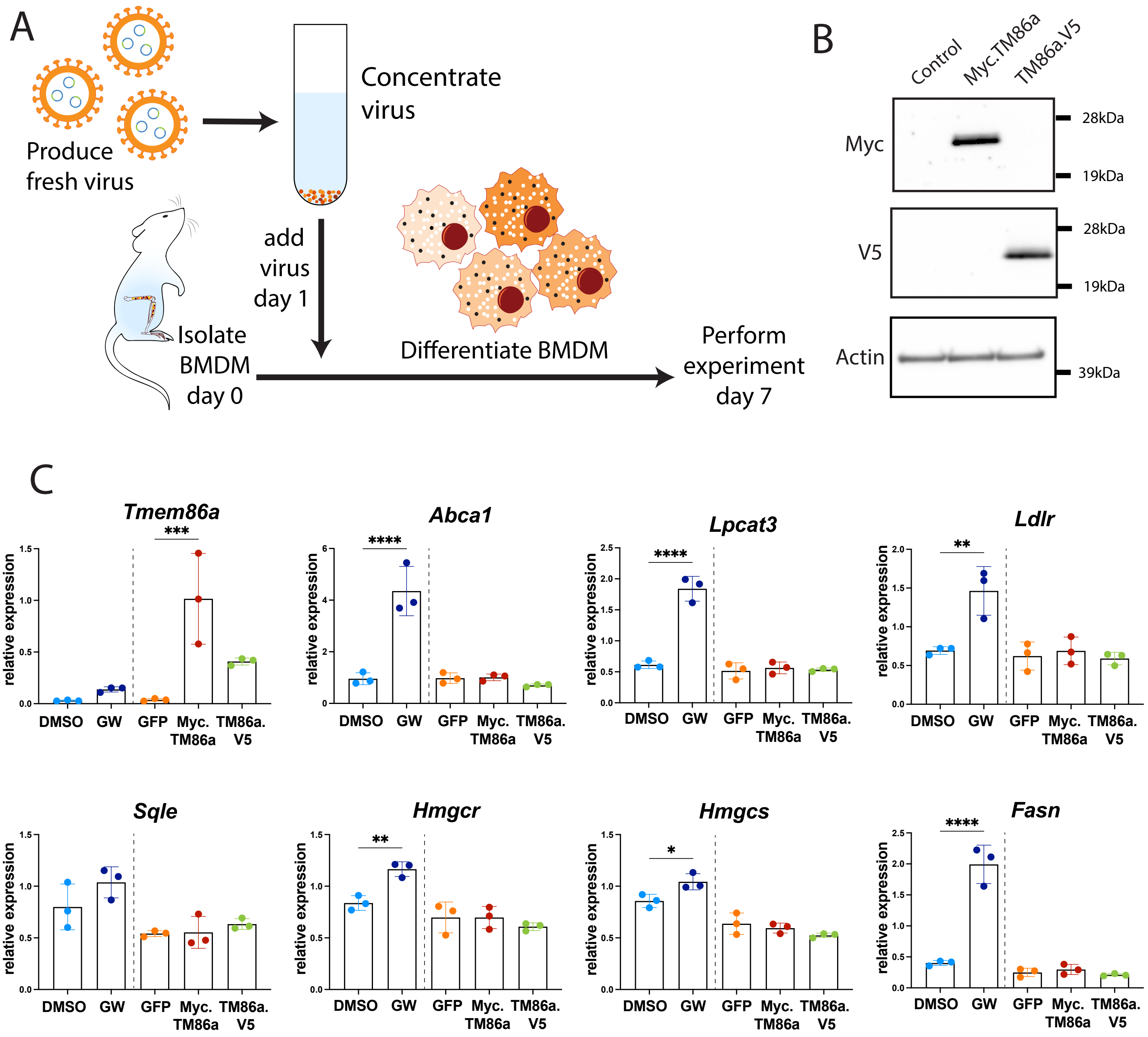

Supplemental figure 6

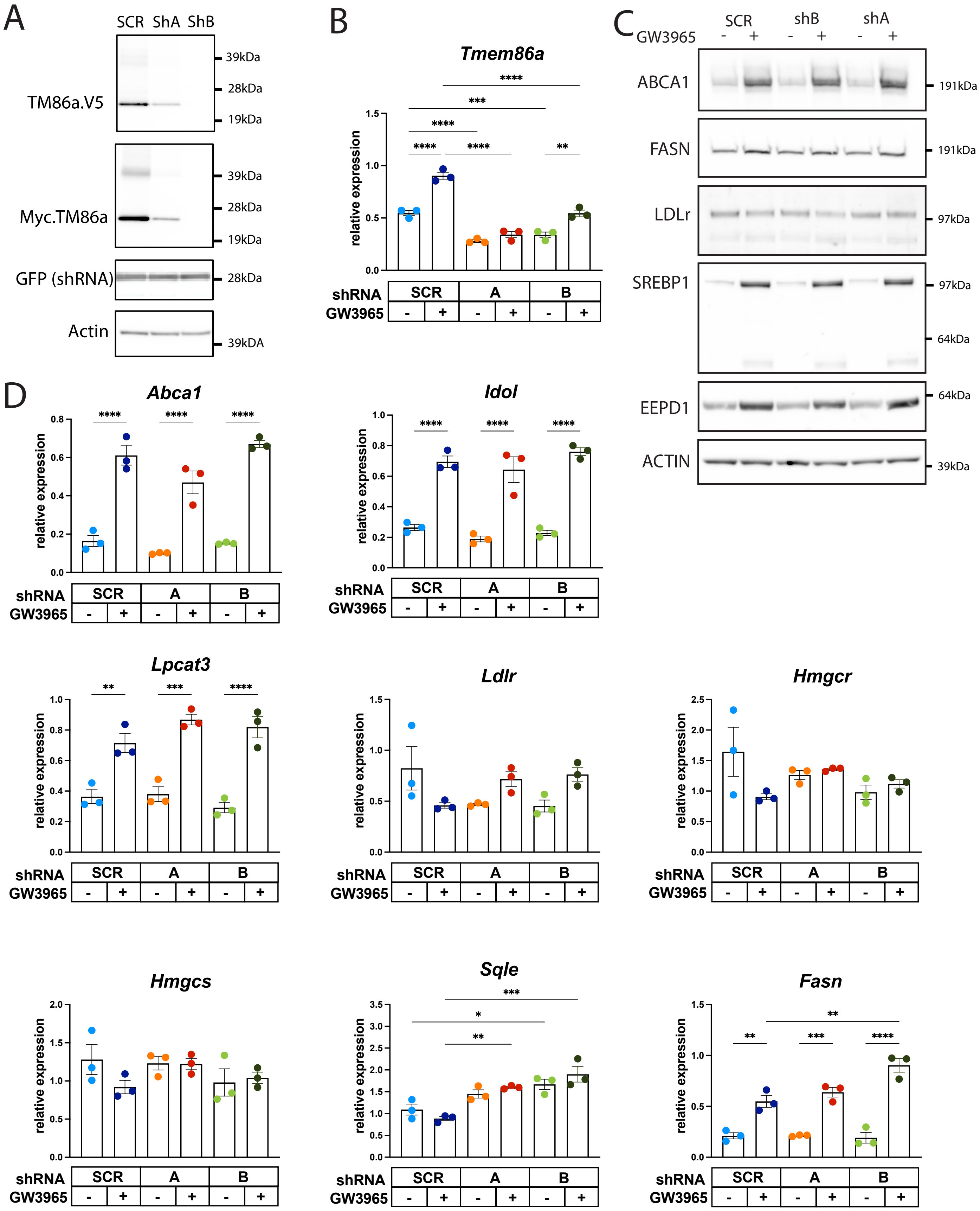

Supplemental figure 7

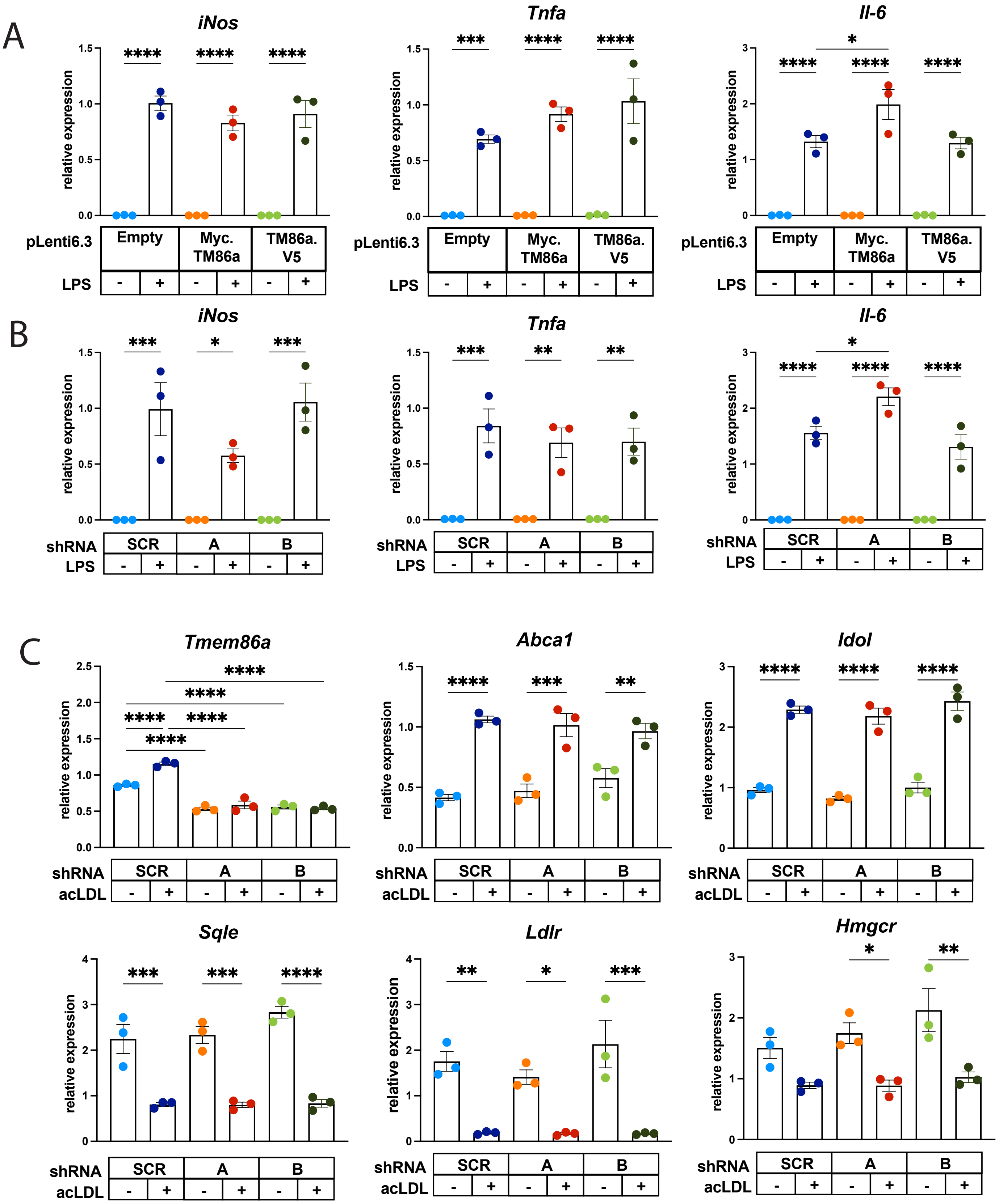

Supplement: Supporting Figures [file mmc1.pdf]
