# Supplementary material for: Sterol-regulated transmembrane protein TMEM86a couples LXR signaling to regulation of lysoplasmalogens in macrophages
Source: J Lipid Res. 2022 Dec 31;64(2):100325. doi: 10.1016/j.jlr.2022.100325 (PMC9926310; doi:10.1016/j.jlr.2022.100325)
Supplement: Supplementary figures legends [file mmc9.docx]

***Supplementary figure legends***

**Supplemental Figure 1. Unbiased lipidomic analysis of LXR stimulated BMDM.**

(***A,B***) BMDM were isolated from wild-type mice (N=5/group) and stimulated with 1µM GW3965 or vehicle control for 16 hr and the cellular lipid composition was measured using global lipidomic profiling. (***A***) Total abundance of the indicated lipid species. (***B***) Total alkyl ether lipids remain unchanged after LXR stimulation. The mean ± SEM are depicted. *p*<0.05, ***p*<0.01. Ceramide (Cer(d), Ceramide-1-phosphate (C1P), Hexosylceramide (HexCer), Dihexosylceramide (Hex2Cer), Bis(monoacylglycero)phosphate (BMP), Alkyldiradylglycerol (DG[O]), Alkyltriradylglycerol (TG[O]), Phosphatidic acid (PA), Phosphatidylcholine (PC), Phosphatidylethanolamine (PE), Phosphatidylglycerol (PG), Lysophosphatic acid (LPA), Lysophosphatidylcholine (LPC), Lysophosphatidylethanolamine (LPE), Lysophosphatidylglycerol (LPG), Phosphatidylinositol (PI), Phosphatidylserine (PS), Sphingosine/Sphinganine 1-phosphate (S1P), Sphingosine, Sphinganine (SPH), Sphingomyelin/Ceramide phosphocholines (SM[d]), Hydroxysphingomyelin (SM[t]).

**Supplemental Figure 2. Expression of *TMEM86a* correlates with that of other lipid-related genes in human plaque macrophages.**

(*left*) Expression of *TMEM86a* was binned ​​into the following groups; Zero: no expression. Low: bottom 25th percentile. Medium: between 25th and 75th percentile. High: Over 75th percentile of *TMEM86a* expression. (*right*) Correlation of binned *TMEM86a* expression with ABCA1, ABCG1 and OLR1.

**Supplemental Figure 3. Characterization of *Tmem86a* expression in murine and human macrophages.**

(***A***) BMDM and thioglycolate-elicited peritoneal macrophages(PMs) were treated as indicated in Figure 3 and expression of *Lpcat3* was measured by qPCR (***B***) BMDM cells were treated with 2µM of GW3965 for 16 hr, and subsequently 5ng/mL LPS was added for 6 hr. Expression of the LXR targets *Eepd1* and *Tmem86a* is shown. (***C***) BMDM cells were cultured in the presence of 50µg/ml acLDL for 24 hr, and expression of the indicated genes was measured by qPCR. (***A,B,C***) Each bar and error are the mean ± SEM of 3 independent experiments. (***D***) *Meta*-analysis of *Tmem86a* expression in thioglycolate-elicited peritoneal macrophages treated with 1µM GW3965 (GW), 1µM T0901317 (T09), and 10µM Desmosterol (Des) for 18 hrs (GSE90815). (***E***) *Meta*-analysis of human PBMC microarray data (GSE25967) stimulated with 1µM T091317 for 24 hr. The expression of *ABCA1* and *TMEM86a* is depicted. Paired t-test was used to compare the values of each human donor. (***F***) PBMCs were isolated and stimulated with 10µM T0901317 for 24 hr. Expression of *ABCA1* and *TMEM86a* was determined by qPCR. Paired t-test was used to compare the values of each human donor. **p*<0.05, ***p*<0.01, ****p*<0.001, *p***** < 0.0001

**Supplemental Figure 4. Characterization of TMEM86a used in *in vitro* lysoplasmalogenase assay.**

(***A***) Schematic representation of the *in vitro* lysoplasmalogenase assay. The release of a fatty aldehyde by a lysoplasmalogen is coupled to its oxidation by yeast alcohol dehydrogenase (ADH) and the cofactor NADH. Oxidation of NADH to NAD+ is monitored and reported (***B***) Absence of ADH abrogates lysoplasmalogenase activity. Each bar and error represent the mean ± SEM change in absorbance at 340 nM of 3 independent experiments. (***C***) Crude membrane fractions of HEK293T cells over-producing human or mouse TMEM86a were isolated and immunoblotted as indicated (N=3). A representative image from one experiment is shown. GM130 was used as a membrane loading control. (***D,E***) Expression constructs for the indicated mutations (green) in the predicted catalytic site were generated, and crude membrane fractions of HEK293T cells over-expressing the indicated TMEM86a construct were immunoblotted as indicated. *** *p* < 0.001.

**Supplemental Figure 5. Gain of *Tmem86a* expression in BMDM does not alter LXR and SREBP signaling.**

(***A***) Schematic representation of the procedure used to transduce BMDM with lentivirus particles. (***B***) BMDM were transduced with the indicated lentivirus-encoding TMEM86a constructs, and total cell lysates were immunoblotted as indicated. A representative image is shown. (***C***) BMDM were treated as in (***B***) or treated with 1µM GW3965 or vehicle for 6 hr and the expression of the indicated genes was determined by qPCR. Each bar and error represent the mean ± SEM from 3 independent experiments. **p*<0.05, ***p*<0.01, ****p*<0.001, *p***** < 0.0001

**Supplemental Figure 6**. **Loss of *Tmem86a* expression in BMDM does not alter LXR and SREBP signaling.**

(***A***) HEK293T were co-transfected with the indicated Myc-TMEM86a or TMEM86a-V5 expression constructs together with 2 independent *Tmem86a*-directed shRNAs or control shRNA (SCRambled) at a ratio of 1:3 for 24 hr, respectively. Total cell lysates were prepared and immunoblotted as indicated. GFP, which is encoded by the shRNA-containing plasmids, was used to control transfection efficiency. (***B***) BMDM were transduced with two independent shRNA constructs and treated with 1 µM GW3965 for 16 hr as indicated. Expression of *Tmem86a* was determined by qPCR. (***C***) BMDM were transduced with the indicated shRNA constructs and subsequently treated with 1µM GW3965 or vehicle control for 16 hr. Total cell lysates were immunoblotted as indicated and a representative image is shown, or (***D***) expression of the indicated genes was determined by qPCR. Each bar and error represents the mean ± SEM of 3 independent experiments. **p*<0.05, ***p*<0.01, ****p*<0.001, *p***** < 0.0001

**Supplemental Figure 7**. **Gain- and Loss-of *Tmem86a* expression does not alter inflammatory gene expression in response to LPS.**

(***A,B,C***) BMDM were transduced with lentiviral particles as described in Supplementary Figures 5,6 to (***A***) overexpress *Tmem86a*, or (***B,C***) silence *Tmem86a* expression, respectively. Subsequently, cells were treated with (***A,B***) 5 ng/mL LPS for 6 hr, or (***C***) 50µg/mL AcLDL for 16 hr. Expression of the indicated genes was determined by qPCR. Each bar and error represents the mean ± SEM of 3 independent experiments. *** *p*<0.001
